# Predicting microcystin concentration action-level exceedances resulting from cyanobacterial blooms in selected lake sites in Ohio

**DOI:** 10.1007/s10661-020-08407-x

**Published:** 2020-07-14

**Authors:** Donna S. Francy, Amie M.G. Brady, Erin A. Stelzer, Jessica R. Cicale, Courtney Hackney, Harrison D. Dalby, Pamela Struffolino, Daryl F. Dwyer

**Affiliations:** 1 U.S. Geological Survey, Ohio-Kentucky-Indiana Water Science Center, 6460 Busch Blvd, Columbus, OH 43229 USA; 2 Lake Erie Center, University of Toledo, Oregon, OH USA

**Keywords:** Cyanobacterial harmful algal blooms, Water quality, Real-time monitoring, Microcystin

## Abstract

Cyanobacterial harmful algal blooms and the toxins they produce are a global water-quality problem. Monitoring and prediction tools are needed to quickly predict cyanotoxin action-level exceedances in recreational and drinking waters used by the public. To address this need, data were collected at eight locations in Ohio, USA, to identify factors significantly related to observed concentrations of microcystins (a freshwater cyanotoxin) that could be used in two types of site-specific regression models. Real-time models include easily or continuously-measured factors that do not require that a sample be collected; comprehensive models use a combination of discrete sample-based measurements and real-time factors. The study sites included two recreational sites and six water treatment plant sites. Real-time models commonly included variables such as phycocyanin, pH, specific conductance, and streamflow or gage height. Many real-time factors were averages over time periods antecedent to the time the microcystin sample was collected, including water-quality data compiled from continuous monitors. Comprehensive models were useful at some sites with lagged variables for cyanobacterial toxin genes, dissolved nutrients, and (or) nitrogen to phosphorus ratios. Because models can be used for management decisions, important measures of model performance were sensitivity, specificity, and accuracy of estimates above or below the microcystin concentration threshold standard or action level. Sensitivity is how well the predictive tool correctly predicts exceedance of a threshold, an important measure for water-resource managers. Sensitivities > 90% at four Lake Erie water treatment plants indicated that models with continuous monitor data were especially promising. The planned next steps are to collect more data to build larger site-specific datasets and validate models before they can be used for management decisions.

**Electronic supplementary material:**

The online version of this article (10.1007/s10661-020-08407-x) contains supplementary material, which is available to authorized users.

## Introduction

The increasing prevalence of cyanobacterial harmful algal blooms (cyanoHABs) and the toxins they produce are a global water-quality issue that threatens human and wildlife health and necessitates additional monitoring of recreational and drinking water source waters (Harke and Gobler 2015; O’Neil et al. 2012). With changes in rainfall and hydrology and increasing temperatures from climate change, preventing and managing cyanoHABs are likely to become more challenging in the future (Paerl et al. 2016). Multiple strategies to address cyanoHABs are ongoing and include reducing nutrient sources, monitoring for and predicting concentrations of toxins, minimizing exposures to humans and animals, and treating waters to reduce or eliminate cyanoHAB toxins once they occur. Identifying monitoring and prediction tools to help make informed decisions on the potential occurrence of harmful levels of toxins in recreational and drinking waters used by the public is an immediate need.

In 2014, the City of Toledo, located in Western Basin of Lake Erie, was forced to issue a do-not-drink advisory due to high concentrations of microcystins found in tap water (Jetoo et al. 2015; Qian et al. 2015). Microcystins, a class of more than 100 cyclic peptide congeners, are one of the most frequently detected freshwater cyanotoxins (Carmichael 1992). To provide warnings of potential cyanoHAB occurrence, area water managers have proactively turned to the HAB Bulletin, a bi-weekly forecast of cyanobacterial density based on remote sensing of cyanobacterial pigments (National Oceanic and Atmospheric Administration--Great Lakes Environmental Research Laboratory 2019). Microcystins, however, are not pigments and cannot be directly detected by remote sensing (Stumpf et al. 2016).

Site-specific predictive models may be used to augment remote sensing based predictions by quantifying the potential for toxin occurrence. These models provide the opportunity to protect the public from exposure to toxins and are based on a variety of factors associated with toxin production. Factors related to bloom formation and (or) toxin concentrations that could potentially be used in models have been previously identified (Joung et al. 2011; Lee et al. 2015; Otten et al. 2012; Wood et al. 2011) including water temperature, concentrations of phosphorus and nitrogen, water turbidity, lake depth, concentrations of toxin and general cyanobacterial genes, and wind direction and speed. High-frequency measurements (several measurements per hour) from optical sensors that measure algal pigments (chlorophyll and phycocyanin) have also shown promise for early-warning systems (Genzoli and Kann 2016; Izydorczyk et al. 2005; McQuaid et al. 2011). A network of water-quality multiparameter instruments has been operating in Lake Erie to measure these pigments, as well as other physical or chemical water-quality parameters such as temperature, pH, specific conductance, and turbidity (Great Lakes Observing System 2019).

In an earlier study at recreational sites in Ohio lakes, measures of the algal community (phycocyanin, cyanobacterial biovolume, and cyanobacterial gene concentrations) and pH were significantly correlated with microcystin concentrations (Francy et al. 2016). Two types of multiple linear regression models could be developed to estimate microcystin concentrations: (1) real-time models that include easily- or continuously-measured factors that do not require a sample to be collected and (2) comprehensive models that use a combination of discrete laboratory-based measurements on samples and real-time factors. Although comprehensive models take more time and effort, they may provide an early warning for and help identify factors associated with microcystin toxin production.

This article describes the results of research by the U.S. Geological Survey (USGS), in cooperation with local and state agencies, to identify factors significantly correlated with microcystin concentrations. Real-time and comprehensive linear regression models were developed to predict an exceedance of a microcystin standard or action value at recreational and water treatment plant sites in Ohio, building on the knowledge gained in a previous study (Francy et al. 2016). Samples and data were collected at six Lake Erie and two inland lake sites with histories of elevated microcystin concentrations (Ohio Environmental Protection Agency 2019). In addition to describing development of models, strategies for validating and using models for management decisions and public notification are discussed.

## Materials and Methods

### Study sites and sampling frequency

**Fig. 1:**
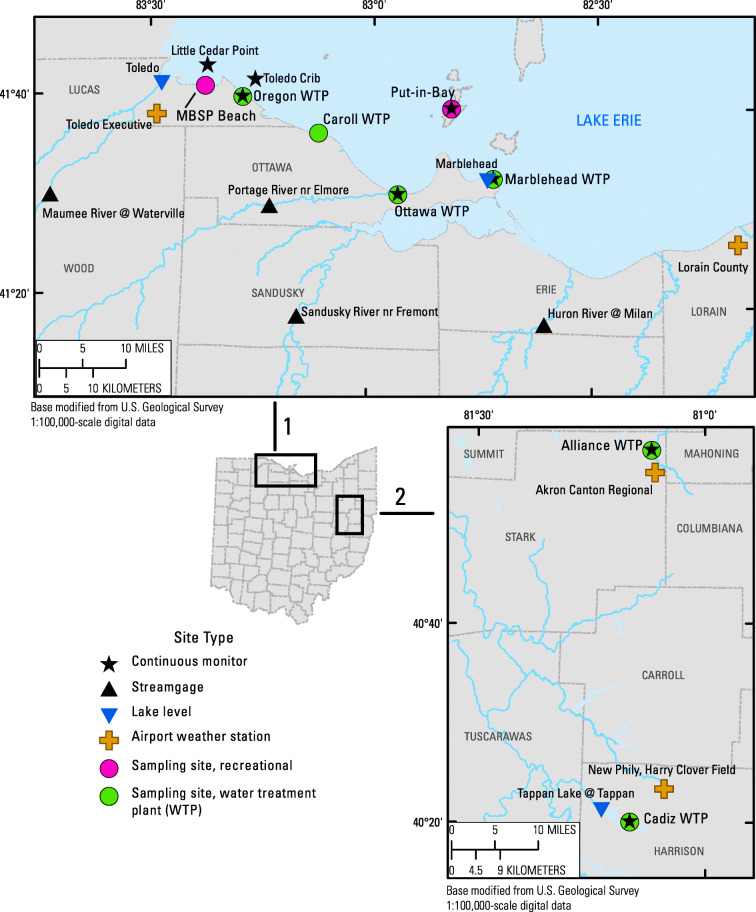
Locations of recreational and water treatment plant sampling sites in Ohio and ancillary data compiled for models, 2016-17 (*MBSP* Maumee Bay State Park)

The study was done at eight locations in Ohio—six in the Western Lake Erie Basin and two in northeast Ohio on inland lakes (Fig. 1). Samples were collected twice a month to twice a week from May to November in 2016–2017, with more frequent sampling during the cyanoHAB season (July–September). At Maumee Bay State Park Lake Erie beach (MBSP Beach), samples were collected during 2013–2014 as part of a previous study (Francy et al. 2015). Site names, official USGS site identification numbers, and agencies collecting and processing samples are listed in Table 1. Samples were collected on predetermined sampling dates, not to target a bloom.

**Table 1:** Study sites and agencies collecting and processing samples at each site in 2016–2017

Site name	USGS station identification number	Agency collecting and processing samples
Recreational sites		
Maumee Bay State Park Beach	414111083223200	University of Toledo
Put-in-Bay	413932082492300	The Ohio State University
Lake Erie water treatment plant (WTP) sites		
Carroll WTP	413642083071100	Carroll Water and Sewer District, University of Toledo
Marblehead WTP	413233082433700	Village of Marblehead, University of Toledo
Oregon WTP	414023083171800	City of Oregon, University of Toledo
Ottawa County WTP	413051082561800	Ottawa County, The Ohio State University
Inland lake water treatment plant sites		
Alliance WTP	405725081070600	City of Alliance, Stark County Health Dept.
Cadiz WTP lower^a^	402003081095301	Stark County Health Dept. or Village of Cadiz
Cadiz WTP upper^a^	402003081095302	Stark County Health Dept. or Village of Cadiz
Cadiz WTP composite^a^	402003081095303	Stark County Health Dept. or Village of Cadiz

Additional samples were collected at Maumee Bay in 2013–2014

*WTP* water treatment plant, *USGS* U.S. Geological Survey

^a^Samples were collected from two spigots (one for the upper and one for lower intake) at the pump station at Tappan Lake. In 2016, the upper and lower intake bottles were analyzed separately; in 2017, the two bottles were composited and analyzed

The study was done at two recreational sites and six water treatment plant sites. MBSP Beach, operated by Ohio State Parks, is in the southwest corner of Lake Erie along Maumee Bay, east of Toledo, Ohio. The Put-in-Bay recreational site is located off South Bass Island in the village of Put-in-Bay, Ohio. Samples were collected offshore near the north side of the island in a semi-enclosed bay frequented by boaters and jet skiers. Four of the water treatment plant (WTP) sites—Oregon, Carroll, Ottawa County (hereinafter “Ottawa”), and Marblehead WTPs—draw water from Lake Erie at intake locations 2.4, 0.3, 0.5, and 0.2 km offshore, respectively; water depths at the intakes were approximately 2.5–7 m. The inland lake water treatment plant sites draw water from Deer Creek Reservoir (Alliance WTP) and Tappan Lake (Cadiz WTP). The Cadiz WTP draws water from two intakes (upper and lower), 1.5 m from the lake bottom in 4.5 m water depths (summer pool level), that are approximately 25 m from the shoreline. The Alliance WTP draws water from a 0.9-m intake at approximately 8-m water depths. Permission was granted by participating water treatment plants to be included in this article.

### Sample collection and field measurements

Samples were collected and analyzed for concentrations of microcystins, cyanobacterial genes, and nutrients, and for phytoplankton community analyses. Sample bottles were pre-washed with non-phosphate detergent, rinsed with tap water, soaked 30 minutes in a 50 mg/L sodium hypochlorite solution, neutralized with 0.05% sterile sodium thiosulfate, dipped in 5% reagent grade hydrochloric acid, and rinsed with sterile deionized water. Before a sample was collected, sample bottles were rinsed three times with native water. If a pump was used to collect a sample, the pump tubing was flushed three times before the sample was collected.

At recreational sites, grab samples were collected approximately 0.3 m below the water’s surface. At MBSP Beach, three 1-L subsamples were collected from cove 3 (a popular swimming area) at 0.7–1.0 m water depths and composited into a 5-L glass bottle. Water temperature, pH, dissolved oxygen, specific conductance, chlorophyll, and phycocyanin (a pigment produced by cyanobacteria) were measured at each subsample location using a hand-held multiparameter instrument calibrated and operated per standard USGS methods (Wilde n.d.) and manufacturer’s instructions (YSI 6-series, YSI Incorporated, Yellow Springs, Ohio). The manufacturer refers to phycocyanin as blue-green algae (BGA) pigment. At Put-in-Bay, samples were collected from the side of a boat by hand using a sterile bottle for cyanobacterial gene analyses and an integrated tube sampler at 0–2 m depths for nutrients, microcystins, algal pigment fluorescence (lab measurement), and phytoplankton community analyses.

At WTP sites, raw water samples were collected from a tap or wet well. At three of the Lake Erie WTP sites (Oregon, Carroll, and Marblehead WTPs), it was not possible to easily collect a sample before a low dose of potassium permanganate (approximately 1 mg/L) was added in the feed intake for mussel control. Permanganate was not used at the inland lake sites. During 2016 at the Oregon WTP, samples were collected from a tap at their low service pump station after permanganate was added; in 2017, samples were collected at the same location after the addition of permanganate was periodically halted for regulatory sampling. At Alliance, Carroll, and Marblehead WTPs, samples were collected from a plant tap. At Ottawa WTP, raw water was collected from the wet well using a bailer with a sterile glass bottle or a submersible pump. At Cadiz WTP, samples were collected from two spigots (one for the upper and one for lower intake) at the pump station at the lake before carbon was added to the wet well. In 2016, the upper and lower intake bottles were analyzed separately; in 2017, the two bottles were composited and analyzed.

Strict quality-assurance and quality-control practices were implemented to ensure collection of accurate, consistent datasets at all sites. Written protocols were distributed to all participating agencies (Table 1). The USGS did several on-site checks of procedures performed by field and laboratory personnel, and any needed corrective actions were taken. In addition to the regular sampling, field quality-control samples were collected and analyzed for all constituents except for phytoplankton community analysis. These included 1 or 2 field blanks and 2 concurrent or sequential replicates per site per year. For quantitative polymerase chain reaction (qPCR) results, if detection occurred in one replicate and not the other, the result from the positive replicate was used; otherwise, an average of two replicates was used for data analysis. Results from quality-control samples were carefully monitored; data were qualified, retests were done, and (or) corrective measures were taken when needed.

### Measurement of microcystins and nutrient concentrations, phytoplankton community composition, and cyanobacterial genes

Depending on the capability of personnel and facilities, samples were processed at a local laboratory or, for some analyses with longer holding times, were shipped to and processed by either the USGS Ohio Water Microbiology Laboratory (USGS OWML) in Columbus, Ohio, or the Ohio Environmental Protection Agency Division of Environmental Services (OEPA DES) in Reynoldsburg, Ohio. Details of sample processing and analytical methods are described elsewhere (Francy et al. 2015).

Samples for total and dissolved nutrients were stored in a dark cooler and processed and preserved within 3 h of sample collection. Processing and analysis for nutrients at Put-in-Bay and Ottawa WTP were done by The Ohio State University Franz Theodore Stone Laboratory (OSU) in Put-in-Bay, Ohio. At all other sites, processing was done by local agencies and shipped to the USGS National Water Quality Laboratory (NWQL) in Denver, Colorado, for analysis. For nutrients analyzed by OSU, water (50 mL) was filtered through a 0.45-μm polycarbonate filter for dissolved nutrients. Approximately 500 mL of whole water for total nutrients and 50 mL of filtrate for dissolved nutrients were frozen until analyses. At OSU, samples were analyzed on a SEAL Analytical QuAAtro continuous segmented flow analyzer using standard methods for nitrate, nitrite, ammonium, and orthophosphate concentrations on filtered samples (EPA 353.1, 353.2, 350.1, and 365.1, respectively) and for total phosphorus and total Kjeldahl nitrogen (TKN) on whole water samples (EPA 365.4 and 351.2, respectively). Total nitrogen concentration was calculated as the sum of TKN, nitrate, and nitrite. For nutrients analyzed by the USGS NWQL, processing procedures were done as per standard USGS methods (Wilde et al. 2002). A four-layer 0.45-μm, 25-mm diameter syringe filter (Tisch Scientific, GD17034) was used to collect 10–20 mL for subsequent analysis of dissolved nutrients. Whole water samples for total nitrogen and total phosphorus analyzed by the USGS were preserved with 1.0 mL of 1:7 sulfuric acid and chilled on ice. Samples were analyzed at the USGS NWQL for concentrations of dissolved nitrite, dissolved nitrate plus nitrite, dissolved ammonia, dissolved orthophosphate, total nitrogen, and total phosphorus per standard USGS methods (Fishman 1993; Patton and Kryskalla 2003; Patton and Kryskalla 2011).

At Put-in-Bay, Ottawa WTP, and Cadiz WTP, a 250-mL aliquot was removed for analysis of phytoplankton abundance and community composition and preserved with 3% Lugol’s iodine. Samples were analyzed for phytoplankton abundance and community composition by BSA Environmental Services, Inc., in Beachwood, Ohio. Phytoplankton slides were prepared using standard membrane-filtration techniques (McNabb 1966; American Public Health Association 1998). A minimum of 400 natural units (colonies, filaments, and unicells) were counted from each sample as described in Lund et al. (1958); counting 400 natural units provides accuracy within 90% confidence limits. In addition, an entire strip filter was counted at high magnification (usually × 630) along with one-half of the filter at a lower magnification (usually × 400) to ensure complete species reporting. Phytoplankton identifications were confirmed by at least two phycologists, and taxonomic nomenclature followed AlgaeBase, a global species database (Guiry and Guiry 2020; Beaver et al. 2013). Biovolume was calculated by using mean measured cell dimensions (Hillebrand et al. 1999).

At Put-in-Bay and Ottawa WTP, samples were analyzed for algal pigment fluorescence using the FluoroProbe benchtop reader (bbe-Moldaenke, Kiel, Germany). The FluoroProbe uses the selective excitation of pigments to partition the signal among four functional phytoplankton groups (green algae, cyanobacteria, diatoms, and cryptophytes; Chaffin et al. 2018b).

Processing and preservation for microcystin analyses were completed within 24 h of sample collection. Two 125-mL high-density polyethylene (HDPE) bottles were triple rinsed with native water, filled with sample, and stored frozen until analysis. Samples were analyzed for total (extracellular and intracellular) microcystins by means of enzyme-linked immunosorbent assay (ELISA) (Microcystins-ADDA ELISA, Abraxis LLC, Warminster, Pennsylvania) by several laboratories per Ohio Environmental Protection Agency (2015). These included the USGS OWML (MBSP Beach and Cadiz WTP samples), Oregon WTP laboratory (Oregon WTP, Carroll WTP, Ottawa WTP, and Marblehead WTP samples), OSU laboratory (Ottawa WTP and Put-in-Bay samples), Alliance WTP laboratory (Alliance WTP samples), and MASI Laboratories in Dublin, Ohio (Cadiz WTP samples). Laboratories are certified by the Ohio Environmental Protection Agency for analysis of total microcystins by ELISA.

All samples for cyanobacterial genes were analyzed at the USGS OWML. At the USGS OWML, aliquots to be analyzed for cyanobacterial genes by qPCR were filtered onto three or four replicate, 0.4-μm pore size Nuclepore polycarbonate filters (Whatman/GE Healthcare, Piscataway, New Jersey) and frozen within 30 h of sample collection. Molecular assays for cyanobacteria associated with microcystin production were done to enumerate (1) general cyanobacteria (16S rRNA); (2) general *Microcystis*, *Dolichospermum*, and *Planktothrix* (16S rRNA); and (3) microcystin toxin genes (*mcyE*) for *Microcystis*, *Dolichospermum*, and *Planktothrix* (Doblin et al. 2007; Ostermaier and Kurmayer 2009; Rantala et al. 2006; Rinta-Kanto et al. 2005; Sipari et al. 2010; Vaitomaa et al. 2003). DNA extraction/purification, standard curve, and limit of detection/quantification calculation procedures are presented elsewhere (Francy et al. 2015); sample inhibition was determined according to procedures in Stelzer et al. (2013). Standard curve and limits of detection and quantification data are listed for the current study (Table S1 in supplemental materials).

In addition to the analyses done by USGS for cyanobacterial genes, samples for cyanobacterial genes were analyzed at OEPA DES as part of regulatory requirements. At the OEPA DES, draft method 705.0 (Ohio Environmental Protection Agency 2016) was followed for filtration, extraction, and qPCR analyses. Differences between USGS OWML and OEPA DES methods include the following: 0.4-μm pore size filter versus 0.8-μm, kit-based DNA extraction/purification versus crude extraction, and molecular assays listed above run in singleplex versus the CyanoDTec assay kit (Phytoxigene™, Akron, Ohio) run in multiplex. CyanoDTec assay results used in this study included a general cyanobacteria (16S rRNA) and microcystin/nodularin toxin gene (“General microcystin *mcyE*”). Standard curve and limits of detection and quantification data are listed in supplemental materials (Table S1). Although results from USGS OWML and OEPA DES were not identical due to the different methods used, concentrations did trend together in a nonlinear relation and were deemed to be comparable, with the USGS OWML reporting higher concentrations.

### Environmental factors

Environmental and water-quality data were compiled for the airport weather station, stream or lake-level gage, and (or) continuous water-quality monitor nearest to the site of interest. These data came from locations that were within 40 km of the study site, and most were within 16 km (Fig. 1). Data definitions and sources are summarized for the current study (Table S2 in supplemental materials). Environmental data were compiled from the National Oceanic and Atmospheric Administration (NOAA), USGS, and (or) The Ohio State University and included rainfall and wind direction and speed (National Oceanic and Atmospheric Administration--National Centers for Environmental Information 2019; USGS 2019a), water levels (National Oceanic and Atmospheric Administration--Tides and Currents 2019; USGS 2019a), daily mean streamflow or gage height (USGS 2019a), and solar radiation (The Ohio State University 2019). Continuous water-quality monitor data collected from multiparameter instruments at Lake Erie sites were obtained from the Great Lakes Observing System (GLOS) HABS Data Portal (GLOS 2019) or for inland lake sites through a private system (WQData Live, NexSens Technology Inc., Fairborn, OH). Site-specific remote sensing satellite data were provided by the National Aeronautics and Space Administration (NASA) from Landsat 8 and reported as mg/m^3^ chlorophyll-*a* (Sandeep Kumar Chittimalli, NASA, written commun., 2018). Chlorophyll-*a* values were reported for the previous day’s measurement or the most recent antecedent data from the satellite.

Downloaded data were checked and transformed by the USGS for use in data analysis and model development. Other agencies coordinated the maintenance of continuous water-quality monitors, which were calibrated twice each year and cleaned periodically from fouling at most sites. For quality assurance, time-series continuous monitor data were plotted by the USGS. Data points in these plots were analyzed in detail and removed if they represented an improbable variation in value when compared to neighboring points or if excessive monitor drift was evident.

Data manipulations for explanatory variables are described elsewhere (Francy et al. 2015) and listed for the current study (Table S2 in supplemental materials). The 24-h averages of continuous monitor measurements up to the approximate time the microcystin sample was collected (i.e., 10 a.m.–10 a.m.) were computed; these 24-h averages were used to calculate averages for 3-, 5-, 7-, and 14-days antecedent to the time of sampling. Rainfall was summed for the 24-h period up to 8 a.m. on the day of sampling to facilitate data compilation from an existing system (USGS 2019b); various multi-day antecedent totals were subsequently calculated. Change in water level was calculated based on the differences between the 10 a.m. water-level value on the date of sampling as compared to the previous day and 7- and 14-day period prior measurement and from the spring average water level. Daily mean streamflow, gage height, and total solar radiation were calculated for the previous day before sampling (midnight to midnight).

### Data management, statistical analysis, and modeling

Daily data for wave heights and field water-quality parameters measured on-site and for turbidity and nutrient, microcystins, and cyanobacterial gene concentrations in discrete samples are available through the USGS National Water Information System database (USGS 2019a) using USGS station identification numbers (Table 1). Data on phytoplankton community composition and datasets used to develop site-specific models are available through data releases (Cicale and Francy 2020; Francy et al. 2020); phytoplankton community analysis data were not used in models.

Data analysis to identify variables significantly correlated to microcystin concentrations and model development were done based on the procedures described in Francy et al. (2016). The factors were segregated based on their potential use in real-time and (or) comprehensive models and whether continuous monitor data were used in model development. Nonparametric correlation coefficients (Spearman’s rho) were calculated to identify associations between microcystin concentrations and other factors. Spearman’s rho measures the strength of the monotonic association between two variables (whether linear or nonlinear) and is resistant to effects of outliers (Helsel and Hirsch 2002). Results from correlation analyses were used to help identify which variables needed to be included for model building, even when multiple data points for the explanatory variable were missing. Multiple minimum reporting limits for cyanobacterial gene concentration data were accommodated in correlation analyses by assigning them a value less than the lowest detection for each assay. Censored nutrient and microcystin data (values below the minimum reporting limit) were assigned one-half the censored value. Scatterplots of key factors were reviewed to ensure relations between other factors and microcystin concentrations were related and were not influenced by one or two outliers.

Additional data analysis and linear regression model development were done with Virtual Beach version 3.07 (U.S. Environmental Protection Agency (USEPA), 2018). Explanatory variables were mathematically transformed as necessary to linearize the relation with the dependent variable. Transformations included log10, inverse, square, square root, and quad root; which one was used (if any) was based on the Pearson’s correlation coefficient (r) between the explanatory variable and microcystin and if an x/y plot of the same indicated improved linearity over the untransformed variable. To identify the best candidate models, models were ranked by a user-selected evaluation criterion such as Predicted Error Sum of Squares (PRESS) or Corrected Akaike Information Criterion (AICC) (Cavanaugh and Neath 2019). Explanatory variables were limited to a maximum variance inflation factor (VIF) of five (to avoid multi-collinearity among explanatory variables), and models were limited to no more than five explanatory variables (due to small sample sizes and to avoid overly complex models). The assumptions associated with ordinary least squares regression required to predict concentrations (Helsel and Hirsch 2002; Chapter 9, Table 9.1) were met. For this study, we were not seeking to predict a variance for the prediction or test hypotheses. Therefore, the two assumptions that had to be met were that (1) the model form is correct and (2) the model is fit with observed and explanatory data that are representative of the range of conditions over which the model will be applied. Model selection and diagnostics were done to meet these assumptions and included tests for statistical significance of explanatory variables and influence and leverage of observations. Tests for influential outliers included Cook’s D, as described in USEPA ( 2018). If a data point was identified as above the critical value for Cook’s D, it was carefully examined and only removed if determined to be erroneous. If a scatterplot between an explanatory variable and the dependent variable indicated a relation was not evident (linear or nonlinear) and (or) was influenced by one or two outliers, the variable was removed and the model selection process was repeated. Finally, a cross-validation step was included in the model selection process to examine the predictive power of all candidate models. This step was done regardless of the model selection criterion used (PRESS is a cross-validation statistic).

Because the models can be used for management decisions and not to explicitly predict a microcystin concentration, the output from each selected model was the probability of exceeding a recommended advisory-level (recreational sites) or action-level (WTPs) microcystin concentration threshold. Model outputs were examined in terms of sensitivity, specificity, and accuracy in estimating concentrations above or below thresholds. The sensitivity is the percentage of exceedances of the advisory or action level that are correctly predicted by the model, the specificity is the percentage of nonexceedances correctly predicted, and the accuracy is the overall percentage of correct responses. A threshold probability was set for each model by examining model-output sensitivities and specificities at different probability levels. The selection of the threshold probability is a compromise between false negative and false positive responses while maintaining a high number of overall correct responses (Francy and Darner 2006). After comparing evaluation criterion for ranked candidate models, the best model was selected based on (1) significance of explanatory variables (*p* < 0.05), (2) sensitivity and specificity to estimate above and below a threshold microcystin concentration, and (3) ability to reasonably explain how each explanatory variable could potentially affect the observed variation in microcystin concentrations.

## Results

### Microcystin concentrations

**Fig. 2:**
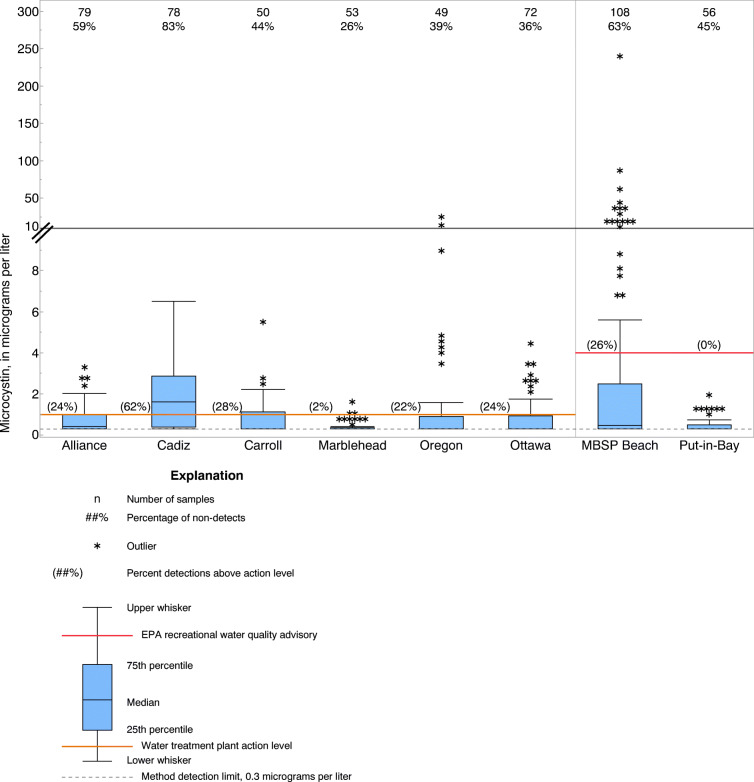
Concentrations of microcystin and percentages of detections at six water treatment plant sites and two recreational sites, 2016–2017 (for Maumee Bay State Park (MBSP) Beach, 2013–2014 data were also included)

Microcystin concentrations, the number of samples, the percent of detections, and the percent of detections above an advisory or action level for each study site are shown in Fig. 2. The action level at recreational sites was based on the USEPA recommended recreational water-quality advisory of 4 μg/L (USEPA 2016). A 1 μg/L threshold was used as practical action level for WTP managers to adjust treatment. At the time of the study (2016–2017), recommended 10-day drinking water health advisories for microcystin were 0.7 μg/L for pre-school-age children and 1.6 μg/L for school-age children through adults (USEPA 2015).

During the study period, the percent of microcystin detections at each site ranged from 26 to 83%, and the highest median microcystin concentration (1.6 μg/L) was found at the Cadiz WTP. Microcystin concentrations found at MBSP Beach ranged from < 0.30 to 240 μg/L, necessitating a split of the *y* axis to adequately view the range of microcystin concentrations. The percent of samples exceeding the recreational or WTP action level ranged from 0% at Put-in-Bay recreational site to 62% at the Cadiz WTP.

### Phytoplankton community analysis

**Fig. 3:**
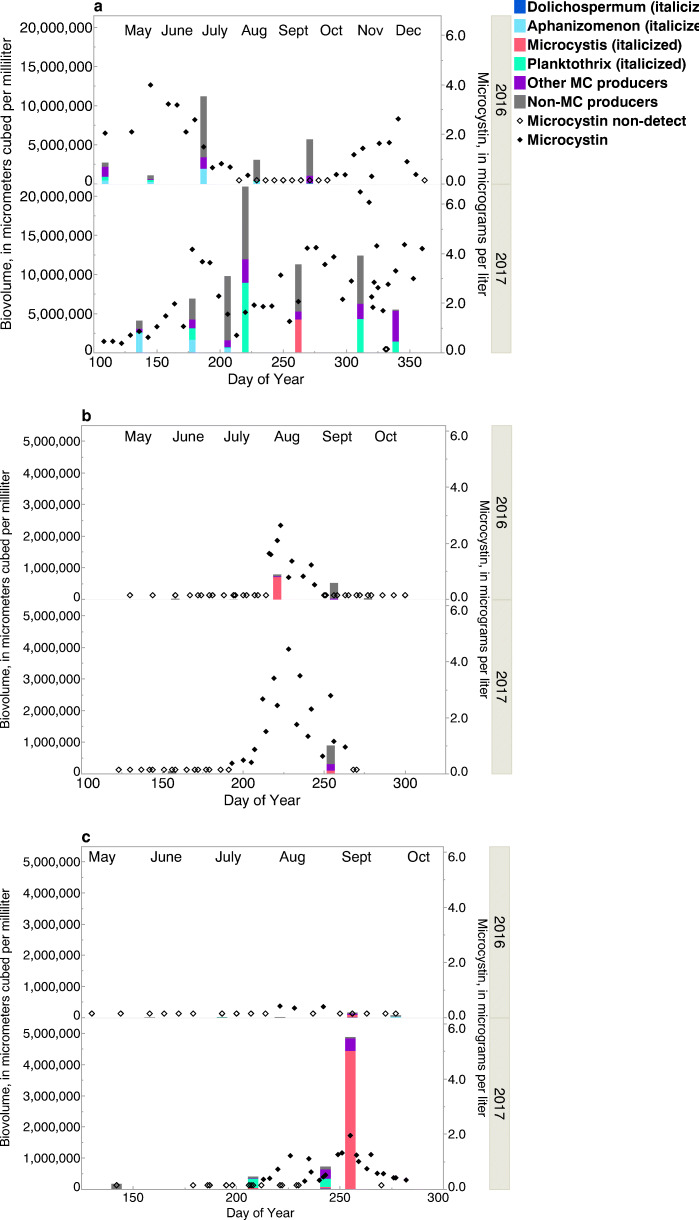
Cyanobacterial biovolumes, relative community compositions of microcystin, and microcystin (MC) concentrations at **a** Cadiz Water Treatment Plant (WTP), **b** Ottawa WTP, and **c** Put-in-Bay recreational site, May–November, 2016–2017

The differences in cyanobacterial community dynamics are shown for three of the study sites—Cadiz WTP, Ottawa WTP, and Put-in-Bay recreational site (Fig. 3). The most common cyanobacterial genera that produce microcystin in freshwaters are shown (*Aphanizomenon, Dolichospermum*, *Microcystis*, and *Planktothrix*), along with other potential microcystin producers (*Aphanocapsa* and *Pseudanabaena*) (USEPA 2014; Bernard et al. 2017). Maximum cyanobacterial biovolume at the Cadiz WTP was 21 times higher than at the Ottawa WTP and four times higher than at Put-in-Bay. At the Cadiz WTP (Fig. 3a), non-microcystin producers were often dominant, even when microcystin was elevated (> 1 μg/L); microcystin concentrations were elevated well into November and December. Microcystin producers present at the Cadiz WTP site included *Aphanizomenon* and other microcystin producers in 2016 (*Pseudanabaena*); in 2017, *Planktothrix* and *Microcystis* were also present*.* At the Ottawa WTP (Fig. 3b), *Microcystis* was dominant when microcystin was elevated (> 1 μg/L) in August 2016 and present with other microcystin producers (*Aphanocapsa*) in September 2017. At Put-in-Bay (Fig. 3c), *Planktothrix* and other microcystin producers (*Aphanocapsa*) were dominant in August 2017 and *Microcystis* was dominant in September 2017 when microcystin was elevated.

### Environmental and water-quality factors

Summary statistics for selected physical, chemical, and cyanobacterial gene results, later used in model development, are shown for each site (Table S3 in supplemental materials). A suite of water-quality measurements made with hand-held instruments were routinely collected at MBSP Beach and Alliance WTP; average values for all measurements were higher at MBSP Beach than at the Alliance WTP. In discrete samples sent to a laboratory, dissolved and total nutrient concentrations were measured at five sites and only total nutrient concentrations at three sites. The highest average concentrations for key nutrient constituents were found at Alliance (ammonia, average = 0.13 mg/L), Ottawa (nitrate plus nitrite, average = 0.60 mg/L), and MBSP Beach (orthophosphate, total nitrogen, and total phosphorus; average = 0.032, 2.65, and 0.12 mg/L, respectively). Average nitrogen to phosphorus (N to P) mass ratios ranged from 11.7 at Cadiz WTP to 44.4 at Put-in-Bay.

A variety of assays were used to quantify cyanobacterial genes by qPCR. General cyanobacteria 16S rRNA was detected in all samples except for two samples at MBSP Beach (98% detected). The highest average concentrations for general *Microcystis* 16S rRNA and *Microcystis*-specific microcystin *mcyE* were found at MBSP Beach and Carroll WTP (> 5.00 log copies/100 mL) and for general *Planktothrix* 16S rRNA and *Plantothrix-*specific microcystin *mcyE* at Alliance WTP and Cadiz WTP (> 6.00 log copies/100 mL). For general *Dolichospermum* 16S rRNA, the lowest average concentration was found at the Ottawa WTP (4.90 log copies/100 mL). The *Dolichospermum-*specific microcystin *mcyE* gene was not found at any site, which agrees with previous research that Lake Erie *Dolichospermum* is not a microcystin-producer (Ouellette et al. 2006). The general microcystin *mcyE* gene was found at all sites where measured (range of detection 39–81%).

Average, minimum, and maximum values for continuous water-quality measurements (24-h antecedent averages) and environmental measurements retrieved from existing sources are shown for selected sites (Table S4 in supplemental materials). This is not an exhaustive list, but rather examples of measurements at Lake Erie and inland lake sites. Parameters measured for at least 2 years were later used in data exploration and model development. Average 24-h phycocyanin measurements were highest at the two inland lake sites (3.6 and 3.7 RFU), and the highest maximum value was found at the Oregon WTP (13.6 RFU). Average pH measurements were the same among the three sites (Oregon, Ottawa, and Alliance WTPs) with pH data presented (average pH = 8.3), with the highest maximum value found at the Oregon WTP (pH = 10). The averages and maximum values for chlorophyll (average 6.0 and max 14.7 RFU) and specific conductance (average 657 and max 815 μS/cm) were highest at the inland lake sites—Alliance WTP and Cadiz WTP, respectively. Environmental data included daily mean streamflow, daily average gage height, lake-level change over 24 h, and rain in the past 24 h.

### Correlations between microcystin concentrations and factors for models

Spearman’s correlation coefficient (rho) was computed to determine the correlation between microcystin concentrations and factors identified as potential explanatory variables for models using data from 2016–2017; 2013–2014 data were also included for MBSP Beach. Factors for real-time models were grouped as follows (Table 2): (a) water-quality hand-held measurements or observations at the site, (b) continuous monitor water-quality measurements, and (c) environmental and seasonal data. Factors for comprehensive models were grouped based on the type of analysis (Table 3): (a) nutrients, (b) algal pigment fluorescence, and (c) cyanobacterial genes. Correlations that were significant at *p* < 0.05 are indicated in the table, negative rho values are in italicized text, and underlined values indicate only 1 year of data collected (Tables 2 and 3).

**Table 2:** Spearman Rank correlation coefficients (rho) between microcystin concentrations and real-time model factors at Ohio recreational and water treatment plant (WTP) sites, 2016–2017

	Recreational sites		Lake Erie WTP sites				Inland lake WTP sites	
Factor	Maumee Bay State Park Beach *n* = 108	Put-in-Bay *n* = 56	Carroll WTP *n* = 50	Marblehead WTP *n* = 53	Oregon WTP *n* = 49	Ottawa County WTP *n* = 72	Alliance WTP *n* = 79	Cadiz WTP *n* = 78
(a) Water-quality hand-held measurements or observations at the site								
Phycocyanin sensor, relative fluorescence units	**0.71**	–	–	–	–	–	NS	–
Chlorophyll sensor, relative fluorescence units	**0.35**	–	–	–	–	–	**0.43**	–
pH	**0.61**	–	–	–	–	–	*0.48*	–
Specific conductance, μS/cm	NS	–	–	–	–	–	NS	**0.34**
Water temperature, deg C	**0.23**	–	NS	–	NS	–	*0.63*	–
Wave height, ft	NS	–	–	–	–	–	–	–
(b) Continuous monitor water-quality, range of rho values for 1-, 3-, 5-, 7-, and 14-day average measurements								
Phycocyanin, relative fluorescence units	**0.28–0.58**	**0.66–0.72**	**0.40–0.77**	**0.36–0.74**	**0.45–0.71**	**0.43–0.81**	NS	**0.35–0.49**
pH	**0.47–0.58**	**0.48–0.53**	**0.53–0.78**	**0.47–0.69**	**0.61–0.70**	**0.63–0.78**	*0.60***–***0.71*	NS**–0.34**
Specific conductance, μS/cm	*0.34***–***0.43*	*0.66***–***0.69*	*0.52***–***0.70*	*0.44***–***0.53*	*0.41***–***0.58*	*0.57***–***0.70*	–	NS
Chlorophyll, relative fluorescence units	NS	**0.32–0.49**	**0.26–0.44**	**0.35–0.50**	**0.27–0.50**	**0.28–0.55**	NS**–0.43**	NS
Temperature, deg C or F	**0.29–0.61**	*0.47***–***0.58*	**0.26–0.32**	NS	NS	NS**–0.29**	*0.86***–***0.80*	NS
Turbidity, NTU	NS**–***0.45*	NS**–0.38**	NS	–	NS**–0.32**	NS**–0.24**	NS**–***0.39*	NS**–0.33**
Oxidative reductive potential, mV	NS**–0.31**	–	NS	*0.41***–***0.71*	NS	NS	NS	–
Dissolved oxygen, mg/L	**0.27–0.44**	NS**–0.27**	NS**–***0.30*	NS	NS	NS	NS**–***0.56*	NS
Solar radiation, Watt/m^2^	NS**–***0.25*	–	–	–	–	–	–	–
Dew point, deg C	NS**–0.44**	–	–	–	–	–	–	–
(c) Environmental and seasonal data								
Gage height, daily average, ft^a^	–	–	–	–	–	–	NS**–0.39**	–
Streamflow, daily mean, cfs^a^	*0.39***–***0.55*	–	*0.34***–***0.65*	NS**–***0.42*	*0.28***–***0.58*	*0.32***–***0.63*	–	–
Rainfall, airport, inches^b^	NS	NS**–***0.27*	NS	NS**–0.34**	NS**–***0.34*	NS**–***0.33*	NS**–0.27**	NS**–0.28**
Rainfall, on-site gage, inches^b^	–	–	–	–	–	–	–	NS**–0.24**
Lake-level change, ft^c^	NS	NS	NS	NS**–***0.32*	NS	NS	–	NS
Wind speed, airport, instantaneous and 24 h, mph	NS	NS	NS**–***0.41*	NS	NS	*0.31***–***0.46*	NS	NS**–0.28**
Wind speed, on-site, instantaneous, mph	–	–	–	–	–	–		NS
Satellite, chlorophyll-a, mg/m^3^	**0.32**	–	**0.31**			–	–	–
Cosine, day of the year	**0.23**	**0.51**	NS	NS	NS	**0.27**	**0.60**	NS
Sine, day of the year	*0.29*	*0.54*	*0.32*	*0.48*	NS	*0.39*	*0.45*	NS

^a^Streamflow and gage height from a nearby river(s) were ranges of coefficients for daily means for 1, 2, or 3 days prior to sampling; 7-day or 14-day peaks and averages

^b^Rainfall amounts were ranges of coefficients for the sums for 1, 2, or 3 days prior to sampling, 48-h and 72-h weighted rainfall amounts, and 7-d and 14-d total rainfall amounts

^c^Lake-level changes from a nearby gage(s) were ranges of coefficients for 1, 7, and 14 days; 7-day average change and 7-d absolute value average change; and change from spring average (April-May)

*n* indicates the number of samples analyzed for microcystin concentrations; underlined values indicate only 1 year of data collected; correlations that were significant at *p* < 0.05 are listed in the table; *NS*, not significant; positive rho values are in bold text; negative rho values are in italicized text; –, the factor was not measured or there were too few measurements; data from 2013–2014 were included for Maumee Bay State Park Beach

**Table 3:** Spearman Rank correlation coefficients (rho) between microcystin concentrations and comprehensive model factors in samples collected at Ohio recreational and water treatment plant (WTP) sites, 2016 − 17

	Recreational sites		Lake Erie WTP sites				Inland lake WTP sites	
Factor	MBSP Beach *n* = 104	Put-in-Bay *n* = 33	Carroll WTP *n* = 33	Marblehead WTP *n* = 17	Oregon WTP *n* = 22	Ottawa County WTP *n* = 32	Alliance WTP *n* = 32	Cadiz WTP *n* = 34
(a) Nutrients, mg/L, lagged up to 2 weeks (unless indicated otherwise)								
Ammonia	NS	NS	–	–	–	NS	**0.40**	*0.29*
Nitrate plus nitrite	*0.25*	NS	–	–	–	*0.45*	NS	**0.43**
Nitrite	*0.26*	NS	–	–	–	*0.43*	**0.46**	NS
Orthophosphate	NS	NS	–	–	–	*0.61*	NS	NS
Total phosphorus	NS	NS	NS	NS	NS	NS	NS	NS
Total nitrogen	NS	NS	NS	NS	NS	NS	**0.45**	NS
Nitrogen to phosphorus ratio, total	NS	NS	NS	NS	NS	*0.31*	NS	NS
Nitrate plus nitrite, unlagged	*0.36*	*0.39*	–	–	–	*0.44*	NS	**0.39**
Orthophosphate, unlagged	NS	NS	–	–	–	*0.60*	NS	NS
(b) Algal pigment fluorescence, μg/L								
Cryptophytes (CryptoFluoro)	–	**0.74**	–	–	–	0.71	–	–
Total fluorescence (TotalFluoro)	–	**0.50**	–	–	–	0.63	–	–
Green algae (GrnFluoro)	–	*0.40*	–	–	–	0.56	–	–
Cyanobacteria (BGFluoro)	–	**0.70**	–	–	–	0.51	–	–
Diatoms (DiaFluoro)	–	NS	–	–	–	NS	–	–
(c) Cyanobacterial genes, log copies per 100 mL, lagged up to 2 weeks								
General Cyanobacteria 16S rRNA	0.29	NS	0.42	NS	**0.51**	NS	NS	NS
General *Microcystis* 16S rRNA	**0.30**	NS	**0.68**	NS	**0.59**	**0.57**	**0.40**	*0.43*
General *Dolichospermum*16S rRNA	–	NS	**0.40**	NS	**0.50**	NS	NS	*0.48*
General *Planktothrix* 16S rRNA	–	NS	NS	NS	NS	NS	**0.45**	**0.55**
*Microcystis*-specific microcystin *mcyE*	**0.42**	NS	**0.79**	0.50	**0.73**	**0.72**	0.73	–
*Planktothrix*-specific microcystin *mcyE*	–	NS	–	NS	–	–	**0.40**	**0.59**
General microcystin/nodularin *mcyE*	–	–	**0.77**	**0.67**	**0.80**	**0.65**	**0.51**	**0.67**

*n* indicates the number of samples analyzed for microcystin concentrations and comprehensive factors; underlined values indicate only 1 year of data collected; correlations that were significant at *p* < 0.05 are listed in the table; NS, not significant; positive rho values are in bold text; negative rho values are in italicized text; –, not measured; data from 2013–2014 were included for Maumee Bay State Park Beach (MBSP Beach)

#### Hand-held measurements or observations at the site

Among measurements made with hand-held multiparameter instruments or observations at the site, significant positive correlations were seen for four parameters measured at the Lake Erie site (MBSP Beach), but significant negative correlations were seen for pH and water temperature at the inland lake site (Alliance WTP) (Table 2a). Water temperature was significantly negatively correlated with microcystin at the Alliance WTP. On closer examination, the highest microcystin concentrations (> 2 μg/L, Fig. 2) at the Alliance WTP were found in late October and November 2016 and 2017 when temperatures were between 7.8 and 14.3 °C, whereas 17 out of 20 samples collected in summer were < 0.30 μg/L when temperatures were > 24.3 °C.

#### Continuous water-quality monitor measurements

Spearman’s correlation coefficients between microcystin concentration and continuous monitor data are presented as ranges of coefficients for 1-, 3-, 5-, 7-, and 14-day average measurements (Table 2b). Correlations between microcystin concentrations and phycocyanin or pH were significant for all time periods at seven out of eight sites; significant correlations were positive except for pH at the Alliance WTP. Negative significant correlations were found for specific conductance at six out of seven sites. Chlorophyll was significantly positively correlated with microcystin for all time periods at five out of eight sites, although significant coefficients were generally lower in magnitude than those found for microcystins with phycocyanin, pH, or specific conductance. The other continuous factors were inconsistently correlated to microcystin concentrations for multiple time periods, significance, and study sites.

**Fig. 4:**
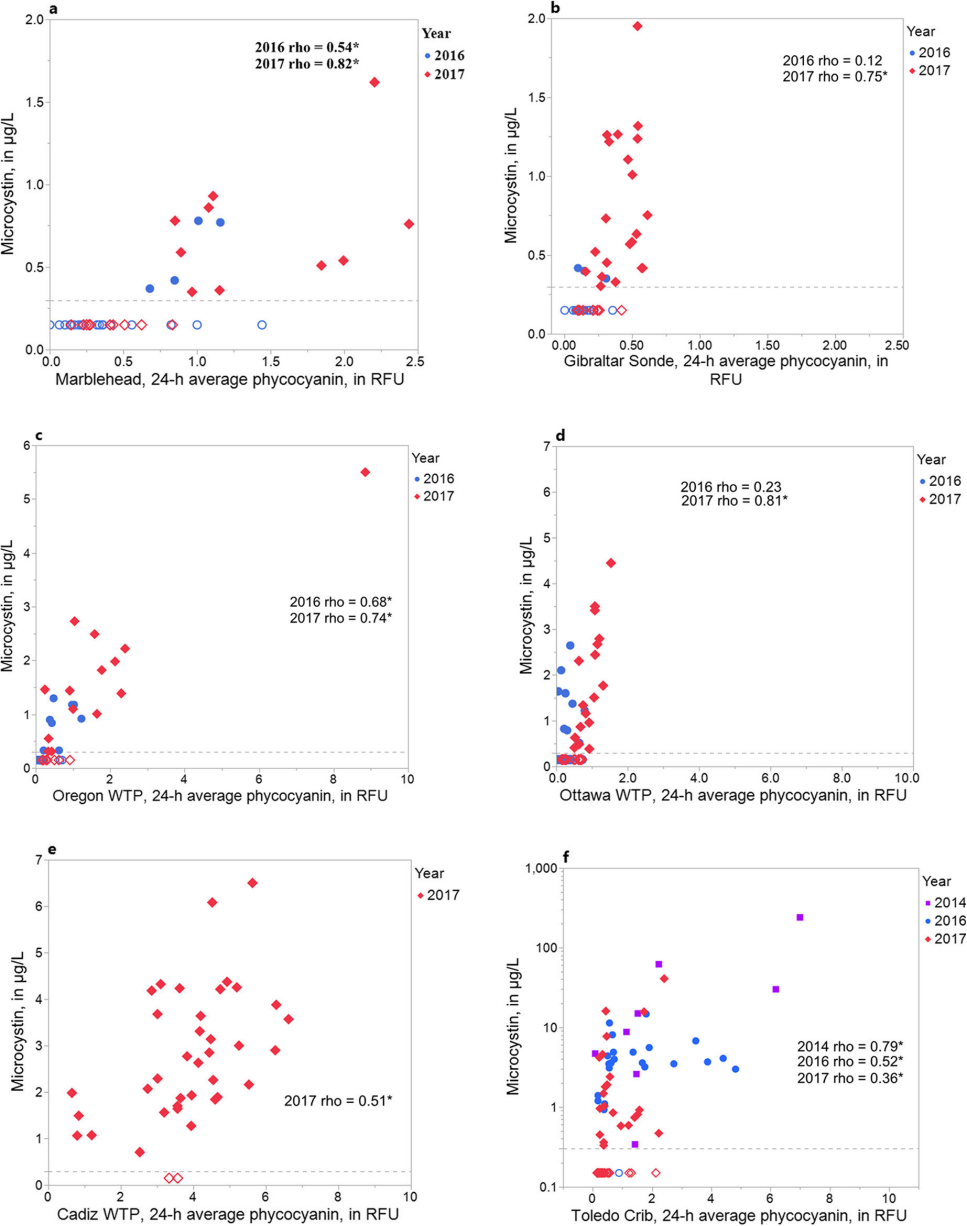
Relations between 24-h average phycocyanin measurements from a continuous monitor to microcystin concentrations, 2016–2017 at **a** Marblehead Water Treatment Plant (WTP), **b** Put-in-Bay recreational site, **c** Carroll WTP, **d** Ottawa WTP, **e** Cadiz WTP, and **f** Maumee Bay State Park (MBSP) Beach. Data from 2014 also included for MBSP Beach; Spearman’s rho coefficients significant at *p* < 0.05 are indicated with an asterisk; dotted line indicates minimum reporting limit, and open circles indicate sample results below minimum reporting limit for microcystin concentrations.

The plotted relations between 24-h average phycocyanin and microcystin concentration are shown in Fig. 4. Overall, the relations were linear with higher microcystin concentrations seen in 2017 than 2016. Measurements obtained from multiparameter water-quality instruments and microcystin concentrations were lowest at the two most eastern Lake Erie sites, Marblehead WTP and Put-in-Bay, with the latter showing smaller phycocyanin to microcystin increases (Figs. 4a and b). At Marblehead WTP and Put-in-Bay recreational site, Spearman’s rho values were statistically significant except for Put-in-Bay during 2016. For Carroll, Ottawa, and Cadiz WTPs, phycocyanin measurements and microcystin concentrations were in a mid-range group (Figs. 4 c–e). At these sites, all Spearman’s rho values were statistically significant except for Ottawa WTP in 2016. For MBSP Beach (Fig. 4f), the 24-h average concentrations of phycocyanin and microcystins were statistically significant in 2014, 2016, and 2017 with the strongest correlation found during 2014.

#### Environmental and seasonal factors

Spearman’s correlation coefficients reported for environmental factors were ranges of coefficients determined for several time periods for gage height, streamflow, rainfall, and lake-level change; one or two coefficients are presented for wind speed and one coefficient for day of the year and satellite data (Table 2c). Streamflow was significantly correlated to microcystin concentrations at all sites where this variable was measured. Rainfall, lake-level change, and wind speed were inconsistently correlated to microcystin concentration for multiple time periods, significance, and study sites. At the two sites where satellite data were available, there were weak but significant positive correlations to microcystin concentrations. A seasonal variable, sine day of the year, was significantly negatively correlated to microcystin concentration at six out of eight sites.

**Fig. 5:**
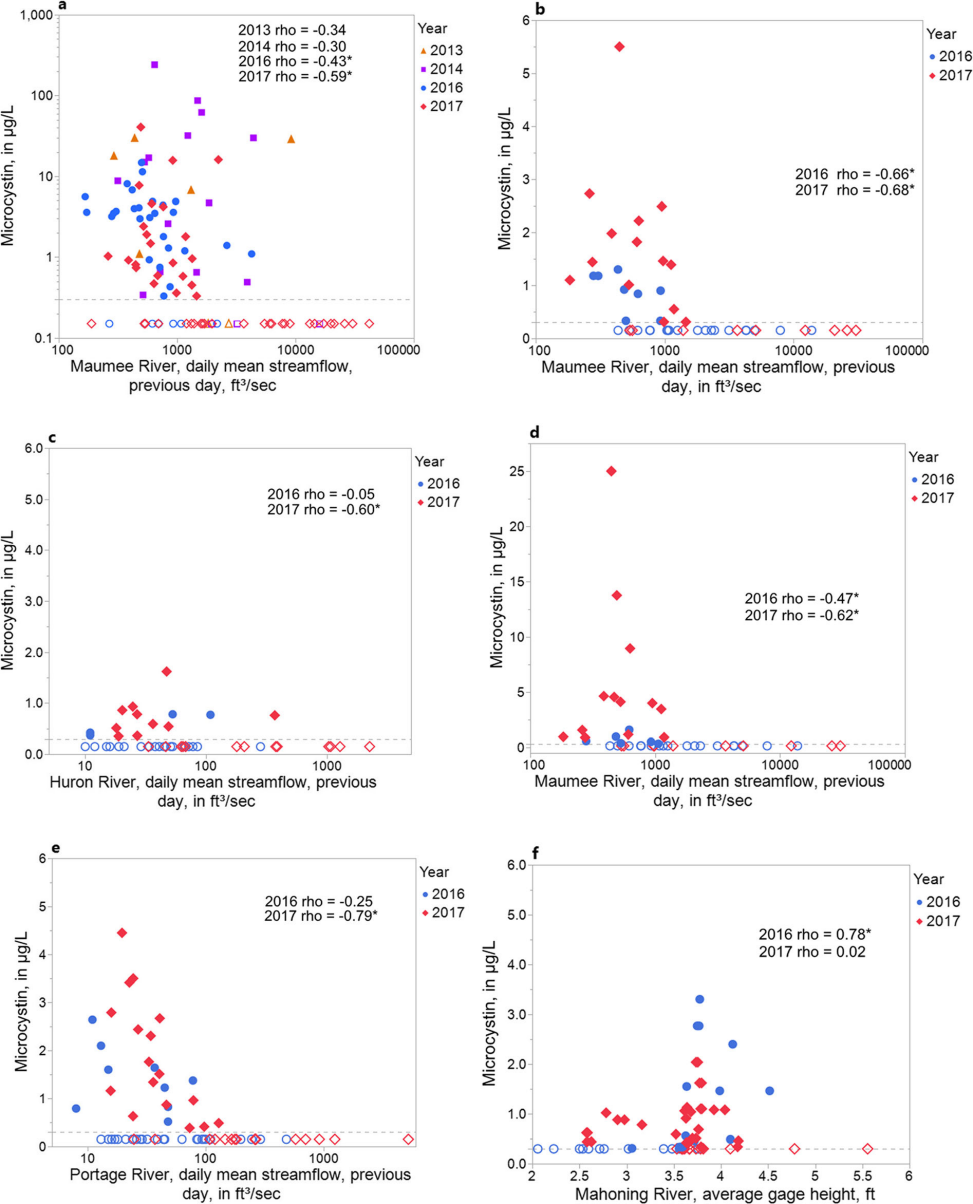
Relations between streamflow or gage height measurements from a nearby river to microcystin concentrations, 2016–2017 at **a** Maumee Bay State Park (MBSP) Beach, **b** Carroll Water Treatment Plant (WTP), **c** Marblehead WTP, **d** Oregon WTP, **e** Ottawa WTP, and **f** Alliance WTP. Data from 2013–2014 also included for MBSP Beach; Spearman’s rho coefficients significant at *p* < 0.05 are indicated with an asterisk; dotted line indicates minimum reporting limit, and open circles indicate below minimum reporting limit for microcystin concentrations.

The plotted relations between daily mean streamflow or gage height (previous day) and microcystin concentrations are shown in Fig. 5. At the five sites with streamflow from a nearby river (Fig. 5a–e), the highest streamflows were associated with microcystin concentrations below detection, and the highest microcystin concentrations were often associated with low streamflows. Streamflow was significantly correlated to microcystin concentration during both 2016 and 2017 at three out of five sites. The relation between gage height and microcystin concentration at the Alliance WTP was statistically significant during 2016, but not during 2017 (Fig. 5f) and was influenced by two outliers (high gage height, < 0.3 μg/L microcystin) in 2017.

#### Comprehensive factors

Comprehensive factors for concentrations of nutrients and cyanobacterial genes were lagged up to 2 weeks, as these were laboratory measurements and were expected to be used in advance of a period of cyanoHAB toxin production (Table 3). At least two of the lagged dissolved nutrients (ammonia, nitrate plus nitrite, nitrite, or orthophosphate) were significantly correlated with microcystin concentration at four out of five sites measured; at Lake Erie sites (Ottawa WTP and MBSP Beach), all significant correlations were negative, whereas at inland lake sites (Alliance and Cadiz WTPs), all significant correlations were positive except for ammonia at Cadiz WTP (Table 3a). Lagged total nitrogen or nitrogen to phosphorus ratios were significant at only one site each. Spearman’s correlations for unlagged nitrate plus nitrite and orthophosphate concentrations with microcystin concentrations were included because continuous real-time instruments are available for these constituents. Unlagged nitrate plus nitrite was significant at four out of five sites; unlagged orthophosphate was significant only at the Ottawa WTP. At the Ottawa WTP and Put-in-Bay, all algal pigment fluorescence measurements were significantly correlated with microcystin concentrations except for diatoms (DiaFluoro) (Table 3b). At least one lagged cyanobacterial gene was significantly correlated with microcystin at all sites except for Put-in-Bay (Table 3c). Strong correlations (rho > 0.70) were found for the *Microcystis-*specific *mcyE* gene at four sites and the general microcystin *mcyE* gene at two sites.

**Fig. 6:**
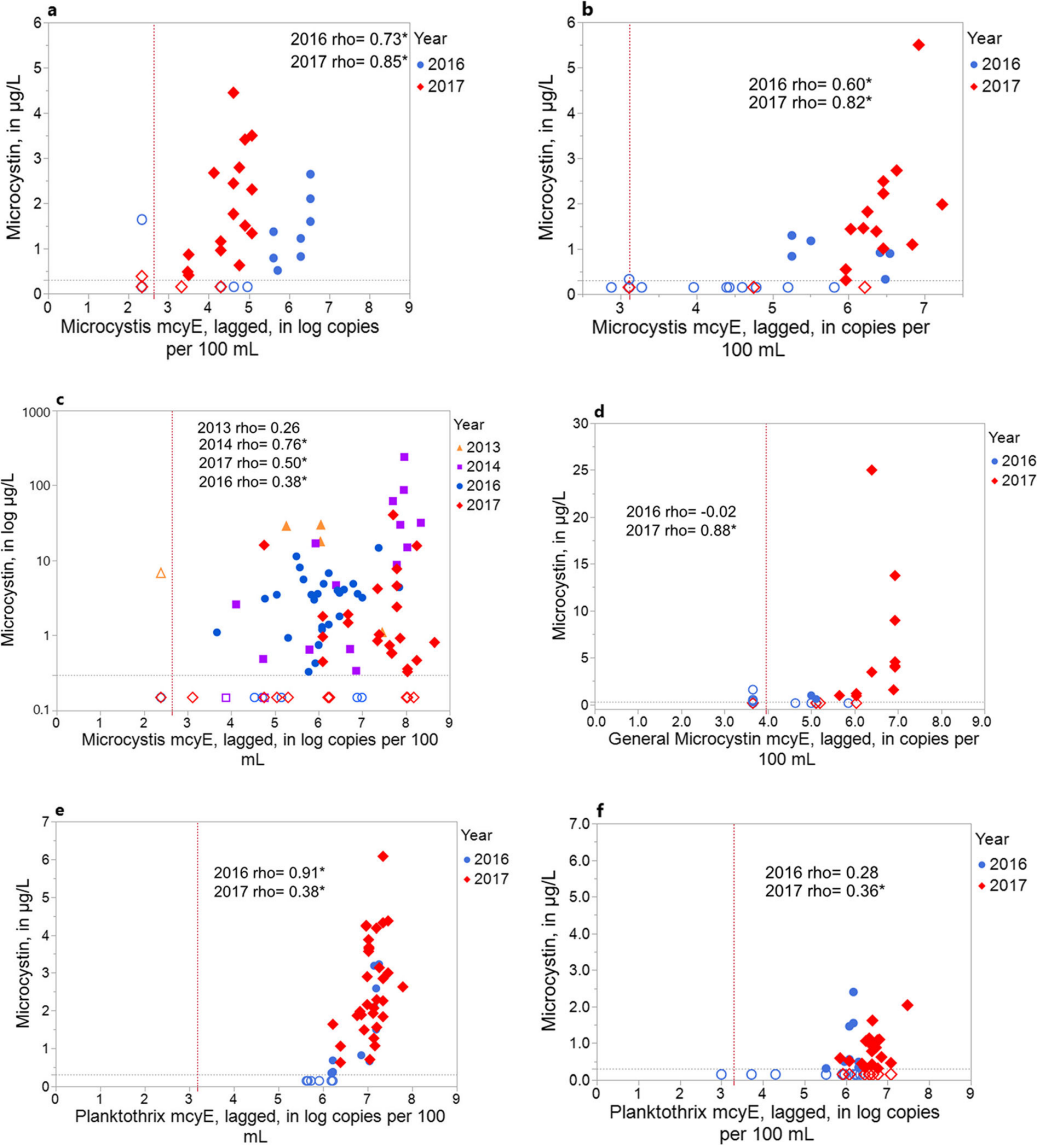
Relations between cyanobacterial gene concentrations (lagged up to 2 weeks) to microcystin concentrations, 2016–2017 at **a** Ottawa Water Treatment Plant (WTP), **b** Carroll WTP, **c** Maumee Bay State Park (MBSP) Beach, **d** Oregon WTP, **e** Cadiz WTP, and **f** Alliance WTP. Data from 2013–2014 also included for MBSP Beach; Spearman’s rho coefficients significant at *p* < 0.05 are indicated with an asterisk; dotted lines indicate minimum reporting limit, and open circles indicate below minimum reporting limit for microcystin and (or) gene concentrations.

The relations between lagged gene concentrations and microcystin concentrations are shown graphically for six sites (Fig. 6). At the Ottawa and Carroll WTPs (Figs. 6 a and b), *Microcystis*-specific *mcyE* concentrations greater than approximately 4.0 and 5.0 log copies/100 mL, respectively, were associated with elevated microcystin concentrations (> 1.0 μg/L). At MBSP Beach (Fig. 6c), however, several samples with microcystin concentrations at or near the minimum reporting limit (0.3 μg/L) had elevated *Microcystis*-specific *mcyE* concentrations. At Oregon WTP (Fig. 6d), the correlation between general microcystin *mcyE* and microcystin concentration was significant during 2017, but not in 2016, owing to lower microcystin concentrations during 2016. At Cadiz and Alliance WTPs (Figs. 6e and f), the relations between *Planktothrix*-specific *mcyE* and microcystin concentration were similar, although microcystin and gene concentrations were lower at the Alliance WTP.

### Models for estimating the probability of exceeding an action threshold for microcystin

Site-specific models were developed to demonstrate the feasibility of using models to estimate exceedance of microcystin concentration thresholds for management decisions. Models were developed for each site when a minimum of 2 years of data were available and were based on real-time variables only (Table 4) and on real-time and comprehensive variables (Table 5). All models included real-time environmental and seasonal variables as these data were available for nearly every day a microcystin sample was collected. Equations for the best models for each site are listed in supplemental materials S5.

**Table 4:**
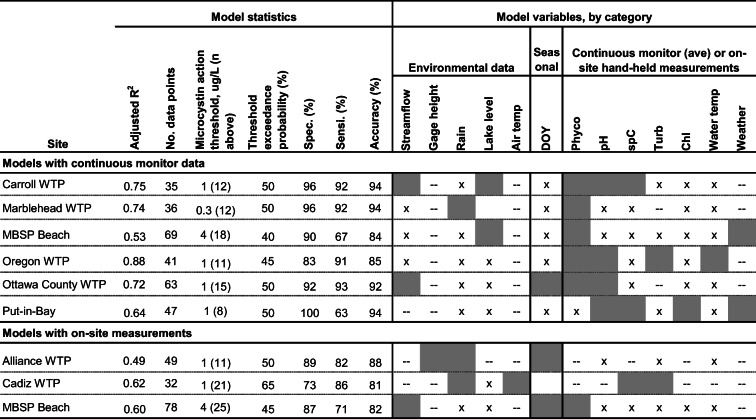
Best models based on real-time variables for estimates of the probability of exceeding a designated action threshold for microcystin

Shading indicates variable class was used in the best model; *x* variable class was used in model development but not included in best model; --, variable class was not available for model development

*WTP* water treatment plant, *MBSP Beach* Maumee Bay State Park Beach, *DOY* day of the year, *Phyco* phycocyanin fluorescence, *spC* specific conductance, *Turb* turbidity, *Chl* chlorophyll

**Table 5:**
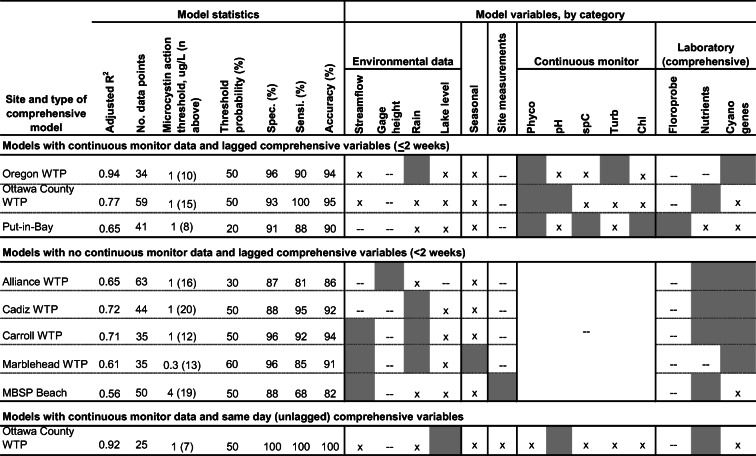
Best models based on comprehensive variables for estimates of the probability of exceeding a designated action threshold for microcystin

Shading indicates variable class was used in the best model; *x* variable class was used in model development but not included in best model; --, variable class was not available for model development

*WTP* water treatment plant, *MBSP* Beach, Maumee Bay State Park Beach, *Phyco* phycocyanin fluorescence, *spC* specific conductance, *Turb*, turbidity, *Chl* chlorophyll

Microcystin thresholds were determined based on potential action levels at each site. For MBSP Beach and Put-in-Bay recreational sites, threshold microcystin concentrations were set at 4 and 1 μg/L, respectively. The threshold concentration at MBSP Beach was based on the USEPA recommended primary contact recreational advisory of 4 μg/L (USEPA, 2016); a lower threshold was used at Put-in-Bay because no samples had microcystin concentrations that exceeded 4 μg/L. At WTP sites, a 1 μg/L threshold was used, except at Marblehead WTP, where a 0.30 μg/L (minimum reporting limit) threshold was used. The 1 μg/L threshold was used as practical action level for WTP managers to adjust treatment; the lower threshold was used at Marblehead WTP because only one sample exceeded 1 μg/L.

#### Real-time models

Real-time models are presented in two categories: (1) models with continuous water-quality data and (2) models without continuous data, having only on-site measurements (Table 4). Adjusted *R*^2^ values for real-time models with continuous monitor data (range 0.53–0.88) were higher than those with on-site measurements (range 0.49–0.62). Threshold exceedance probabilities for action established for each model ranged from 40–65%. Sensitivities > 90% were achieved in models for the four Lake Erie WTPs (Carroll, Marblehead, Oregon, and Ottawa), 80–90% for the inland lake WTPs (Alliance and Cadiz), and < 80% for the recreational site models. At the Alliance and Cadiz WTPs, models were developed with only on-site measurements because only 1 year of continuous monitor data were available. Specificity of the Cadiz WTP model (73%) was lower than models for the other sites because a large percentage of the samples (21 out of 32) were above the 1 μg/L action threshold at Cadiz WTP. At MBSP Beach, data were available to develop both types of models. The real-time on-site model for MBSP Beach had a slightly higher adjusted *R*^2^ than the real-time continuous monitor model (0.60 and 0.53, respectively); however, specificity, sensitivity, and accuracy were equivalent for both models.

Environmental data were used in all real-time models except for the Oregon WTP and Put-in-Bay models, and continuous monitor data were used in all models except for the Alliance WTP model. Phycocyanin fluorescence was used in six models, pH and streamflow or gage height in four models, and rainfall, season, and specific conductance in three models. Other variables were used in one or two site-specific models. The MBSP Beach and Put-in-Bay continuous monitor models included weather variables available from the Toledo Crib buoy (wind speed and dew point) and Gibraltar Island buoy (wind speed), respectively (Table S2).

#### Comprehensive models

Comprehensive models (those with laboratory measurements) are presented in three categories (Table 5): (1) models with continuous monitor data and lagged comprehensive variables, (2) models with no continuous monitor data and lagged comprehensive variables, and (3) a model with continuous monitor data and same-day (unlagged) comprehensive variables. The adjusted *R*^2^ values for comprehensive models with continuous monitor data (range 0.65–0.94) were generally higher than those for models with no continuous monitor data (range 0.56–0.72). Threshold probabilities ranged from 20 to 60%. Sensitivities > 90% were achieved in five WTP models (Oregon, Ottawa (2), Cadiz, and Carroll), 80–90% for three models (Put-in-Bay, Alliance WTP, and Marblehead WTP), and < 80% for the MBSP Beach model.

Three comprehensive models were developed that had both continuous monitor and comprehensive variables. The comprehensive variables included general Microcystin *mcyE* genes for Oregon WTP, nitrogen to phosphorus ratio for Ottawa WTP, and total fluorescence at Put-in-Bay (Table S3). Among these three comprehensive models, the Oregon WTP model had the highest *R*^2^ value (0.94) and the Ottawa WTP model had the highest sensitivity (100%). The models with no continuous monitor data were those without 2 years of data available (Alliance WTP and Cadiz WTP) or those in which continuous monitor data had to be purposely excluded for comprehensive variables to be used in models (Carroll WTP, Marblehead WTP, and MBSP Beach). These models, with nutrient (dissolved or N to P ratios) and (or) cyanobacterial gene variables, would only be used if continuous monitor data were not available. The MBSP Beach model included site measurements of turbidity and wave height. At the Ottawa WTP, a model with continuous monitor data and same-day comprehensive variables was developed that included orthophosphate as a variable.

## Discussion

### Factors that could be used in models

In an earlier study (Francy et al. 2016), factors that could be used in models to estimate microcystin levels at recreational sites were identified and models were developed for one site, MBSP Beach, using a small dataset (*n* = 24). In the current study, WTP sites, larger datasets, and modeling at eight sites expanded on this earlier work. The sites included the recreational beach investigated during the earlier study, a boater swim area of a Lake Erie island, four WTP sites in the Western Lake Erie Basin, and two inland lake WTP sites. The percentages of microcystin detections ranged from 26 to 83% at the sampling sites during 2016–2017 (MBSP Beach included data from 2013–2014). Data were collected or compiled on factors to be used in real-time and comprehensive models. Factors for real-time models are those that are available in real-time for management decisions, including those from hand-held and continuous water-quality measurements and environmental and seasonal data. Factors for comprehensive models include those that require that a sample be collected and analyzed in a laboratory.

As a first step in model development, correlation coefficients (Spearman’s rho) were computed between microcystin concentrations and real-time and comprehensive factors. This exploratory data analysis provides insights into factors that may potentially be used in multiple linear regression models. The significance and direction (positive or negative) of significant correlations for some key variables were different between Lake Erie and inland lake sites. These included phycocyanin, pH, specific conductance, and nutrient concentrations. The best site-specific real-time and comprehensive models were then developed when at least 2 years of data were available. In addition to model statistics, it was important to be able to reasonably explain how each explanatory variable could potentially affect the observed variation in microcystin concentrations.

Among real-time factors, phycocyanin fluorescence, pH, specific conductance, streamflow or gage height, and a seasonal factor (sine of day of the year) were significantly correlated to microcystin concentrations at most sites (Table 2). These were the variables commonly used in real-time models, with phycocyanin used most often. Phycocyanin is a light-harvesting pigment protein produced by cyanobacteria, and the concentration of phycocyanin is often used as a proxy for cyanobacterial biomass (Humbert and Törökné 2016). However, because phycocyanin is a measurement of both non-toxin- and toxin-producing cyanobacteria and phycocyanin has high intracellular variability (Stumpf et al. 2016), caution should be taken in using phycocyanin alone to estimate microcystin concentrations. After phycocyanin, pH and streamflow or gage height were most often used in real-time models. High pH (pH > 9) is partially caused by cyanobacteria while it also enhances their dominance (Jacoby et al. 2000). High biomasses of cyanobacteria use bicarbonate (HCO_3_^-^) as a carbon source, which releases hydroxide (OH^-^) and increases pH. Specific conductance had a positive effect on one of the inland lake models. During the summertime, elevated specific conductance is associated with low lake levels and stagnation, which are conditions that favor cyanobacterial growth (Andres et al. 2019). The inclusion of specific conductance as a negative variable in Lake Erie models is harder to explain, but it could simply reflect the temporal patterns of cyanobacteria (highest late summer) and specific conductance (lowest late summer). Specific conductance is proportional to major ion concentration (Wetzel 2001), and the seasonal decline of specific conductance may be due to decreases in calcium and carbonate as the result of growth of the invasive bivalve *Dreissena* mussel. Streamflow was often negatively significantly correlated to microcystin concentrations. Lower streamflows occur during periods with high evapotranspiration and less turbulent waters, conditions conducive to cyanobacterial dominance. Bertani et al. (2017) found a negative relation between bloom size and streamflow and suggested this was consistent with cyanobacterial growth being favored by lower summer flushing rates and higher residence time. In the current study, a seasonable variable was used in three models. Graham et al. (2017) also included a seasonal variable (cosine day of the year) in models for microcystin occurrence in a Kansas reservoir. The seasonal variable reflects the consistent seasonal pattern in microcystin occurrence. The further importance of a seasonal variable was shown in the current study, where microcystin was negatively correlated to water temperature at an inland lake site because microcystin concentrations were higher in the fall when temperatures were low. Although wind speed and lake-level change were inconsistently correlated among sites and different timeframes for the same site, these variables were used in two models. The influence of wind direction and lake-level change on microcystin concentrations is complex. *Microcystis* growth is favored during periods with high water stability; however, short wind-induced mixing events may have a positive effect on cyanobacterial growth by enhancing resuspension of nutrients (Bertani et al. 2017).

Many of the real-time factors were averages over time periods antecedent to the time the microcystin sample was collected. Average conditions over larger time periods may be more important to the development of current microcystin concentrations than are conditions at the time of sampling. Bertani et al. (2017) used three time-lagged variables (2, 8, and 30 days for wind velocity and stress, irradiance, temperature, and streamflow) in models to estimate cyanobacterial bloom size in the Western Lake Erie Basin. They stated that different time scales help to minimize collinearity among variables. In another study in the Western Lake Erie Basin, Chaffin et al. (2018b) indicated that one should average continuous monitor data over 1 h or 24 h to get a better correlation with water-quality data than one measurement made at the time a sample is collected. They stated that one measurement of phycocyanin, for example, could be influenced by large spikes of *Microcystis* colonies drifting past the sonde.

Comprehensive factors investigated included nutrient and cyanobacterial gene concentrations and algal pigment laboratory measurements. Comprehensive data for nutrients and cyanobacterial genes were lagged up to 2 weeks to provide an advanced warning of elevated microcystin concentrations.

Some lagged dissolved nutrients showed significant but weak correlations to microcystin concentrations whereas lagged total nitrogen or phosphorus were seldom significantly correlated to microcystin concentrations (Table 3). In Lake Erie, the highest concentrations of nutrients occur during the spring or early summer when water temperatures are too low to support cyanobacterial blooms (Chaffin et al. 2011). Total N to P mass ratios were significantly correlated to microcystin concentrations at one Lake Erie site, but used in models at several sites. Other investigators (Jacoby et al. 2015) found that the best predictor of microcystin concentration categories in nine lakes in Washington, USA, was N to P ratio. The authors stated that low N to P ratios favor cyanobacterial dominance because many cyanobacteria genera have lower cellular N to P requirements than other phytoplankton. However, a 10-year data set of 246 lakes showed that middle N to P ratios had the highest probability of microcystins present and the highest concentrations of microcystins (Scott et al. 2013). Some sites had correlations (either positive or negative) between ambient concentrations of nitrate, nitrite, ammonia, and orthophosphate (either on the 2-week lag or unlagged) with microcystins, but there was no consistent pattern of these correlations. Because snapshot grab samples may not adequately account for complex biogeochemical processes, one option may be to collect continuous nutrient data. In the current study, unlagged nitrate plus nitrite and unlagged orthophosphate concentrations were significantly correlated to microcystin concentrations at four sites and one site, respectively. This is important for future management decisions, because it is possible to measure these constituents *in situ* continuously in real-time and obtain more than a snapshot by use of a probe or automatic analyzer. While nitrate concentration by itself was not a good predictor of microcystin concentration in this study, nitrate concentrations > 0.2 mg N/L indicate the potential for microcystins to be present because cyanobacteria cannot produce high levels of microcystins in nitrogen-limited waters (Chaffin et al. 2018a). The same-day orthophosphate concentration was used as a negative variable in one model. *Microcystis* has been shown to grow well under low orthophosphate conditions, facilitated by a high-affinity orthophosphate uptake system (Gobler et al. 2016). In addition, low orthophosphate concentrations may result from increased metabolic activity during warmer months from cyanobacteria and other organisms. It is challenging, however, to interpret orthophosphate concentrations because cyanobacteria can store enough P intracellularly for several cellular division cycles (Baldia et al. 2007; Gobler et al. 2016). Because the roles of nutrients in development of cyanobacterial blooms and toxins are complex (Srivastava et al. 2016), the use of nutrients and the interaction between nutrients and other factors in models to estimate toxin concentrations need to be further investigated.

Significant Spearman’s correlations between cyanobacterial genes (lagged) and microcystin concentrations were found at all sites and were used in several models. Microcystin concentrations have been found to correlate with copy numbers of toxin genes *mcyA* (Srivastava et al. 2012) and *mcyE* (Otten et al. 2012), and *mcyE* and *mcyA* (Conradie and Barnard 2012), genes required for microcystin toxin production. In laboratory studies, higher nitrogen concentrations resulted in increased microcystin concentrations and increased expression of the *mcy* genes (Chaffin et al. 2018a; Harke and Gobler 2015; Srivastava et al. 2016).

### Towards an operational system using models for management decisions

Because these models can be used for management decisions, important measures of model performance are sensitivity, specificity, and accuracy in terms of estimates above and below an action threshold. Sensitivity is especially important in that managers want to err on the side of caution to predict exceedance of the action threshold. Providing an exact estimate of the microcystin concentration is not as important, as the models are only one tool for assessing current water-quality conditions. On that note, sensitivities > 90% at four Lake Erie WTPs indicated that models with continuous monitor data were especially promising (Table 4). The importance of servicing sondes to obtain reliable data in the future is important. The USGS recommends that continuous monitors be calibrated and cleaned for fouling as often as needed based on-site conditions and data quality objectives (Wagner et al. 2006). In Western Lake Erie Basin waters, maintenance functions on sondes are typically performed once a month or more often if needed (Erin Bertke, U.S. Geological Survey, oral commun.).

At several sites, continuous monitor data had to be intentionally excluded for lagged comprehensive data to be included in the best models. This means that real-time factors may be sufficient at some sites and that comprehensive factors would only be needed in the event continuous monitor data are not available. At one site, a comprehensive model with orthophosphate measured at the time of sampling and continuous monitor data provided 100% sensitivity and specificity. With further research and data collection, employing an automatic analyzer for measuring orthophosphate and nitrate concentrations may prove to be useful. Collecting data on comprehensive factors, however, may be worth the extra time and effort at some sites to provide an advanced warning of a microcystin toxin event. This includes continuing to collect samples for analysis of total nutrients, calculating N to P ratios at some sites, and measuring algal pigment fluorescence in a local laboratory. Assays targeting the microcystin toxin gene representing the dominant strain or general microcystin production may be useful predictors of future toxic blooms.

It should be noted that the relations between explanatory variables and microcystin concentrations were sometimes different among the 2 years investigated during this study (3 or 4 years at MBSP Beach). Indeed, biovolumes and community profiles (including the genera of microcystin producers) were different in 2016 and 2017 at the three sites where phytoplankton community analysis samples were collected (Fig. 3). Microcystin concentrations were lower in 2016 than in 2017 at most sites. Nevertheless, consistent significant correlations among sites, years, and time periods for some variables and high model performance statistics (sensitivities, specificities, and accuracies) show promise in using models for management decisions.

If the models prove to be valuable management tools after validation, they can be used to trigger sample collection, adjust treatment options at WTPs, and provide real-time advisories at recreational sites. The models developed during this study are not intended for immediate use by beach and WTP managers but are rather exploratory work to demonstrate how models could be used for future management decisions. More data need to be collected to build site-specific datasets and validate models before they can be practically applied. Indeed, explanatory variables and microcystin concentrations vary site by site and indicate there are complexities that still need to be understood. The model results are not intended to be used as a surrogate for microcystin concentrations—direct measurement of the toxin is still required. Finally, a system for compiling data and running the models daily would be a valuable tool for water-resource managers. The Great Lakes NowCast (USGS 2019b) has been providing real-time estimates of *Escherichia coli* based on models since 2014. The NowCast system provides speed and efficiency for managers to manage data and develop and validate models. It can be easily modified to include cyanotoxins.

## Conclusions

The ability to quickly estimate the probability of exceeding an action threshold for microcystin concentrations is valuable to recreational site and WTP managers. In this study, we showed that site-specific multiple linear regression models with accuracies > 80% could be developed for Great Lakes and inland lake sites that use a variety of water-quality and environmental factors related to microcystin concentrations. Real-time models commonly included variables such as phycocyanin, pH, specific conductance, and streamflow or gage height. Many of the real-time factors were averages over time periods antecedent to the time the microcystin sample was collected, including water-quality data compiled from continuous monitors. Sensitivities > 90% at four Lake Erie WTPs indicated that models with continuous monitor data were especially promising. Comprehensive models (those which have data from discrete samples analyzed in a laboratory) were useful at some sites with lagged variables for cyanobacterial toxin genes, dissolved nutrients, and (or) N to P ratios. More work needs to be done to validate models before they can be applied for management decisions.

## Electronic supplementary material

ESM 1 (DOCX 24 kb)

ESM 2 (XLSX 30 kb)
