# Supplementary material for: Predicting microcystin concentration action-level exceedances resulting from cyanobacterial blooms in selected lake sites in Ohio
Source: Environ Monit Assess. 2020 Jul 14;192(8):513. doi: 10.1007/s10661-020-08407-x (PMC7360538; doi:10.1007/s10661-020-08407-x)
Supplement: Supplementary file 1 — (DOCX 24 kb) [file 10661_2020_8407_MOESM1_ESM.docx]

**Supplemental S5**. Best models based on real-time and comprehensive variables for estimates of the probability of exceeding a designated action threshold for microcystin (Models are organized in alphabetic order by site)

Sources of data are provided in Table S2.

# Alliance Water Treatment Plant

## Real-time model with on-site measurements

Microcystin_ugL = -1.223 + 0.4386*(QUADROOT(Cosine_date)) + 0.04252*(SQUARE(Sum14d_precip_CAK)) + 5.324*(INVERSE(GHt14dpeak_Mahoning)) + 0.0446*(SQUARE(GHtdm2_Mahoning))

WHERE

Microcystin_ugL is the concentration of microcystin in micrograms per liter. A threshold of 1 μg/L was used for this model.

Cosine_date is the cosine of the day of the year, calculated as 2πD/365. The quad root (4th root) of this variable was used.

Sum14d_precip_CAK is the total rainfall, in inches, for the 24-hour period up to and including 8 a.m. on the day of sampling, summed for the last 14 days, Akron-Canton Airport. This variable was squared.

GHt14dpeak_Mahoning is the maximum average daily gage height, in feet, during the past 14 days, Mahoning River at Alliance (USGS 03086500), The inverse of this variable was used.

GHtdm2_Mahoning is the average daily gage height, in feet, 2 days before the day the microcystin sample was collected (midnight to midnight), Mahoning River at Alliance (USGS 03086500). This variable was squared.

## Comprehensive model with no continuous monitor data and lagged comprehensive variables (< 2 weeks)

Microcystin_ugL = -0.5892 - 0.0134*(SQUARE(GHt30dpeak_Mahoning)) + 0.01798*(QUADROOT(LAG_Plank_mcyE_cp100mL)) + 14.94*(LAG_Nitrite_mgL) + 0.04667*(SQUARE(GHtdm2_Mahoning))

WHERE

Microcystin_ugL is the concentration of microcystin in micrograms per liter. A threshold of 1 μg/L was used for this model.

GHt30dpeak_Mahoning is the maximum average daily gage height, in feet, during the past 30 days, Mahoning River at Alliance (USGS 03086500), The square of this variable was used.

LAG_Plank_mcyE_cp100mL is the concentration of the *Planktothrix mcyE* gene, in genomic copies per microliter, in a discrete sample collected <2 weeks before the microcystin sample was collected. The quad root of this variable was used.

LAG_Nitrite_mgL is the concentration of nitrite, in milligrams per liter, determined by a laboratory in a discrete sample collected <2 weeks before the microcystin sample was collected.

GHtdm2_Mahoning is the average daily gage height, in feet, 2 days before the day the microcystin sample was collected (midnight to midnight), Mahoning River at Alliance (USGS 03086500). This variable was squared.

# Cadiz Water Treatment Plant

## Real-time model with on-site measurements

Microcystin_ugL = -0.4945 - 0.2176*(Turb_NTRU) - 0.8967*(SQUARE(Rain48W_in_PHD)) - 0.1242*(TPN_TempAir_C_max_DM1) + 0.01108*(SpCond_uScm)

WHERE

Microcystin_ugL is the concentration of microcystin in micrograms per liter. A threshold of 1 μg/L was used for this model.

Turb_NTRU is the turbidity, in nephelometric turbidity ratio units, measured with a portable turbidimeter.

Rain48W_in_PHD is the rainfall, in inches, cumulated sums for the past 48 hours, giving the most weight to the most recent value, as follows: (2 * Dm1) + Dm2. DM1 is the 24 hour total up to 8 a.m. on the day the microcystin samples was collected; DM2 is the rain 2 days ago, Henry Clever Field Airport in New Philadelphia, Ohio. This variable was squared.

TPN_TempAir_C_max_DM1 is the air temperature, in degrees Celsius, maximum value for the previous day (midnight to midnight), Weather Station at Tappan Lake 402120081134200.

SpCond_uScm is the specific conductance, in microsiemens per centimeter, measured by use of a portable meter.

## Comprehensive model with no continuous monitor data and lagged comprehensive variables (< 2 weeks)

Microcystin_ugL = -1.247 + 1.259*(LOG10(OEPA_Micro_GCuL_LAG2)) - 1.684*(RainD_1_PHD) + 0.5772*(DIFF(LOG10(GenPlank_cp100mL_LAG2),LOG10(GenDoli_cp100mL_LAG2))) + 0.1264*(NtoP_ratio_LAG2)

WHERE

Microcystin_ugL is the concentration of microcystin in micrograms per liter. A threshold of 1 μg/L was used for this model.

OEPA_Micro_GCuL_LAG2 is the concentration of a general cyanobacteria (16S rRNA) microcystin/nodularin toxin gene (*mcyE*), in genomic copies per microliter, in a discrete sample collected <2 weeks before the microcystin sample was collected. This variable was log_10_ transformed.

RainD_1_PHD is the total rainfall, in inches, for the 24-hour period up to and including 8 a.m. on the day of sampling, Henry Clever Field Airport in New Philadelphia, Ohio.

(DIFF(LOG10(GenPlank_cp100mL_LAG2),LOG10(GenDoli_cp100mL_LAG2))) is the difference of (1) the log concentration of the General *Planktothrix* gene, in genomic copies per microliter, minus (2) the log concentration of the General *Dolichospermum* gene, in genomic copies per microliter, in a discrete sample collected <2 weeks before the microcystin sample was collected.

NtoP_ratio_LAG2 is the total nitrogen to total phosphorus ratio, determined by a laboratory in a discrete sample collected <2 weeks before the microcystin sample was collected.

# Carroll Water Treatment Plant

## Real-time model with continuous monitor data

Microcystin_ugL = 4.672 - 0.6162*(LkLevelChg24_ft_Marble) + 0.05989*(PROD(Leorgn_PH_Ave24hr,Leorgn_BGA_RFU_Ave24hr)) - 0.5647*(LOG10(MR_Dis_dm1)) - 0.008903*(Leoc_SpCond_Ave7d)

WHERE

Microcystin_ugL is concentration of microcystin in micrograms per liter. A threshold of 1 μg/L was used for the model.

LkLevelChg24_ft_Marble is the change in lake level over the past 24 hours calculated as today’s lake level for 10:00 a.m. (a typical sampling time) minus yesterday’s lake level at 10:00 a.m., NOAA Marblehead 9063079.

PROD(Leorgn_PH_Ave24hr,Leorgn_BGA_RFU_Ave24hr) is the product of (1) pH, measured by the Oregon WTP continuous monitor, average of 24 hours up to and including 10 a.m., the time the microcystin sample was collected and (2) the phycocyanin (also called blue-green algae) in relative fluorescence units, measured by the Oregon WTP continuous monitor, average of 24 hours up to and including 10 a.m., the time the microcystin sample was collected.

MR_Dis_dm1 is the daily mean streamflow, in cubic feet per second, for the previous day (midnight to midnight), USGS Maumee R at Waterville 04193500. This variable was log_10_ transformed.

Leoc_SpCond_Ave7d is the specific conductance, in microsiemens per centimeter, measured by the Ottawa WTP continuous monitor, averaged over the previous 7 days up to and including 10 a.m. on the day microcystin sample was collected.

## Comprehensive model with no continuous monitor data and lagged comprehensive variables (< 2 weeks)

Microcystin_ugL = -0.5096 + 0.2455*(SUM(LOG10(GenDoli_cp100mL_LAG),LOG10(Micro_mcyE_cp100mL_LAG))) - 12.24*(INVERSE(NtoP_ratio_LAG)) - 0.3721*(TDZ_Sum7d_precip) - 0.0001169*(MR_Dis_30dAve)

WHERE

Microcystin_ugL is the concentration of microcystin in micrograms per liter. A threshold of 1 μg/L was used for this model.

SUM(LOG10(GenDoli_cp100mL_LAG),LOG10(Micro_mcyE_cp100mL_LAG)) is the sum of (1) the log concentration of the General *Dolichospermum* 16S rRNA gene, in genomic copies per 100 milliliters, and (2) the log concentration of the *Microcystis*-specific microcysin *mcyE* gene, in genomic copies per 100 milliliters, in a discrete sample collected <2 weeks before the microcystin sample was collected.

NtoP_ratio_LAG is the total nitrogen to total phosphorus ratio, determined by a laboratory in a discrete sample collected <2 weeks before the microcystin sample was collected. The inverse of this variable was used.

TDZ_Sum7d_precip is the total rainfall, in inches, for the 24-hour period up to and including 8 a.m. on the day of sampling, summed for the last 7 days, Toledo Executive Airport.

MR_Dis_30dAve is the daily mean streamflow, in cubic feet per second, for the 24-hour period for the previous day before sampling, averaged over 30 days. Maumee River at Waterville OH (USGS site 04193500).

# Marblehead Water Treatment Plant

## Real-time model with continuous monitor data

Microcystin_ugL = 0.1787 - 0.07399*(LPR_Rain_sum14d) + 1.46*(TDZ_AirportRain48_in) + 0.2877*(Marble_BGARFU_Ave24hr)

WHERE

Microcystin_ugL is concentration of microcystin in micrograms per liter. A threshold of 0.3 μg/L (minimum reporting limit) was used for the model.

LPR_Rain_sum14d is the total rainfall, in inches, for the 24-hour period up to and including 8 a.m. on the day of sampling, summed for the last 14 days, Lorain County Regional Airport.

TDZ_AirportRain48 _in is the total rainfall, in inches, for the 24-hour period up to and including 8 a.m. on the day of sampling, lagged one day (2 days ago), Toledo Executive Airport.

Marble_BGARFU_Ave24hr is the phycocyanin (also called blue-green algae) in relative fluorescence units, measured by the Marblehead WTP continuous monitor, average of 24 hours up to and including 10 a.m., the time the microcystin sample was collected.

## Comprehensive model with no continuous monitor data and lagged comprehensive variables (< 2 weeks)

Microcystin_ugL = 0.3218 + 0.1692*(Cosine_date) + 1.806*(TDZ_AirportRain48_in) - 0.0007045*(HuronR_dm3) + 0.04399*(OEPA_Micro_GCuL_LAG)

WHERE

Microcystin_ugL is the concentration of microcystin in micrograms per liter. A threshold of 0.30 μg/L was used for this model.

Cosine_date is the cosine of the day of the year (D), calculated as 2πD/365.

TDZ_AirportRain48_in is the total rainfall, in inches, for the 24-hour period up to and including 8 a.m. on the day of sampling, lagged one day (2 days ago), Toledo Executive Airport.

HuronR_dm3 is the daily mean streamflow, in cubic feet per second, 3 days prior to the day the microcystin sample was collected (midnight to midnight), USGS Huron R at Milan 04199000.

OEPA_Micro_GCuL_LAG is the concentration of a general cyanobacteria (16S rRNA) microcystin/nodularin toxin gene (*mcyE*), in genomic copies per microliter, in a discrete sample collected <2 weeks before the microcystin sample was collected.

# Maumee Bay State Park recreational site

## Real-time model with continuous monitor data

Log10 (Microcystin_ugL) = -2.04 + 0.2317*(TolCrib_BGA_RFU_Ave5d) + 0.2428*(TolCrib_WSPD_ms_Ave7d) + 0.06532*(TolCrib_DEWPT_Ave5d) + 0.197*(LkLevelChg7day)

WHERE

Microcystin_ugL is concentration of microcystin in micrograms per liter, log_10_ transformed for modeling. A threshold of 4 μg/L (minimum reporting limit) was used for the model.

TolCrib_BGA_RFU_Ave5d is the phycocyanin (also called blue-green algae) in relative fluorescence units, measured by the Toledo Crib continuous monitor (Site 45165), averaged over the previous 5 days up to and including 10 a.m. on the day microcystin sample was collected.

TolCrib_WSPD_ms_Ave7d is the wind speed, in meters per second, measured by the Toledo Crib continuous monitor (Site 45165), averaged over the previous 7 days up to and including 10 a.m. on the day microcystin sample was collected.

TolCrib_DEWPT_Ave5d is the dew point, in degrees Celsius, measured by the Toledo Crib continuous monitor (Site 45165), averaged over the previous 5 days up to and including 10 a.m. on the day microcystin sample was collected.

LkLevelChg7day, in feet, is the change in lake level calculated as today’s lake level for 10:00 a.m. (a typical sampling time) minus lake level at 10:00 a.m. 7 days ago, NOAA Toledo 9063085.

## Real-time model with on-site measurements

LOG10(Microcystin_ugL) = 0.6367 - 0.2366*(LOG10(Dis_dm3)) - 0.3151*(Cosine_date) + 1.651e-05*(Phyco_cellsML)

WHERE

Microcystin_ugL is the concentration of microcystin in micrograms per liter. A threshold of 4 μg/L was used for this model. This variable was log_10_ transformed.

Dis_dm3 is the daily mean streamflow, in cubic feet per second, for the 24-h period (midnight to midnight) 3 days before the microcystin sample was collected, USGS Maumee R at Waterville 04193500. This variable was log_10_ transformed.

Cosine_date is the cosine of the day of the year (D), calculated as 2πD/365.

Phyco_cellsML is the phycocyanin (also called blue-green algae), in cells per milliliter, measured at the site with hand-held multiparameter water-quality instrument at the time the microcystin sample was collected.

## Comprehensive model with no continuous monitor data and lagged comprehensive variables (< 2 weeks)

LOG10(Microcystin_ugL) = 0.2894 - 1.187*(SQUAREROOT(WaveHt_ft)) + 0.01073*(Turb_NTRU) - 5.207e-05*(Dis_7dAve) + 0.1613*(Nox_mgL_LAG1)

WHERE

Microcystin_ugL is the concentration of microcystin in micrograms per liter. A threshold of 4 μg/L was used for this model. This variable was log_10_ transformed.

WaveHt_ft is the wave height, in feet, measured with a yardstick at the site at the approximate same time the microcystin sample was collected. The square root of this variable was used.

Turb_NTRU is the turbidity, in nephelometric turbidity ratio units, measured with a portable turbidimeter from a sample collected at the same time the microcystin sample was collected.

Dis_7dAve is the average of daily mean streamflow, in cubic feet per second, for the past 7 days, USGS Maumee R at Waterville 04193500.

Nox_mgL_LAG1 is the nitrate plus nitrite concentration, in milligrams per liter, determined by a laboratory in a discrete sample collected <2 weeks before the microcystin sample was collected.

# Oregon Water Treatment Plant

## Real-time model with continuous monitor data

Log10 (Microcystin_ugL -1) = -2.246 + 0.005944*(Leorgn_TURB_NTU_Ave5d) + 0.09333*(Leorgn_BGA_RFU_Ave3d) + 0.2365*(Leoc_pH_Ave3d) + 0.01444*(Leoc_WaterTemp_C_Ave5d)

WHERE

Microcystin_ugL is the concentration of microcystin in micrograms per liter. For model development the log10 of the microcystin concentration +1 was used to avoid an outlier with high influence in the model. A threshold of 1 μg/L was used for the model.

Leorgn_TURB_NTU_Ave5d is the turbidity in nepholometric turbidity units, measured by the Oregon WTP continuous monitor, averaged over the previous 5 days up to and including 10 a.m. on the day microcystin sample was collected.

Leorgn_BGA_RFU_Ave3d is the phycocyanin (also called blue-green algae) in relative fluorescence units, measured by the Oregon WTP continuous monitor, averaged over the previous 3 days up to and including 10 a.m. on the day microcystin sample was collected.

Leoc_pH_Ave3d is the pH, measured by the Ottawa WTP continuous monitor, averaged over the previous 3 days up to and including 10 a.m. on the day microcystin sample was collected.

Leoc_WaterTemp_C_Ave5d is the water temperature in degrees Celsius, measured by the Ottawa WTP continuous monitor, averaged over the previous 5 days up to and including 10 a.m. on the day microcystin sample was collected.

## Comprehensive model with continuous monitor data and lagged comprehensive variables (< 2 weeks)

MicrocystinPlus1_log_ugL = 0.05204 + 0.1248*(LOG10(OEPA_Micro_GCuL_LAG)) - 0.04045*(TDZ_Rain_sum14d) + 0.007891*(Leorgn_TURB_NTU_Ave24hr) + 0.08832*(Leorgn_BGA_RFU_Ave3d)

MicrocystinPlus1_log_ugL is the concentration of microcystin in micrograms per liter. A threshold of 1 μg/L was used for this model. This variable was transformed by adding 1.0 ug/L to each value and taking the log_10_.

OEPA_Micro_GCuL_LAG is the concentration of a general microcystin/nodularin toxin gene (mcyE), in genomic copies per microliter, in a discrete sample collected <2 weeks before the microcystin sample was collected. This variable was log_10_ transformed.

TDZ_Rain_sum14d is the rainfall, in inches, daily values summed for the last 14 days, based on the 24-hour period up to and including 8 a.m. on the day of sampling, Toledo Executive Airport.

Leorgn_TURB_NTU_Ave24hr is the turbidity in nepholometric turbidity units, measured by the Oregon WTP continuous monitor, averaged over the past 24 hours up to and including 10 a.m. on the day microcystin sample was collected.

Leorgn_BGA_RFU_Ave3d is the phycocyanin (also called blue-green algae) in relative fluorescence units, measured by the Oregon WTP continuous monitor, averaged over the previous 3 days up to and including 10 a.m. on the day microcystin sample was collected.

# Ottawa County Water Treatment Plant

## Real-time model with continuous monitor data

Microcystin_ugL = -10.17 + 1.572*(Leoc_BGA_RFU_Ave24hr) + 1.406*(Leoc_pH_Ave24hr) - 0.8684*(LOG_10_(Portage_Dis_30dPeak)) - 1.371*(Cos_DOY)^2^

WHERE

Microcystin_ugL is the concentration of microcystin in micrograms per liter. A threshold of 1 μg/L was used for this model.

Leoc_BGA_RFU_Ave24hr is the phycocyanin (also called blue-green algae) in relative fluorescence units, measured by the Ottawa WTP continuous monitor, average of 24 hours up to and including 10 a.m., the time the microcystin sample was collected.

Leoc_pH_Ave24hr is the pH, measured by the Ottawa WTP continuous monitor, average of 24 hours up to and including 10 a.m., the time the microcystin sample was collected.

Portage_Dis_30dPeak is the maximum daily mean discharge, in cubic feet per second, during the past 30 days, Portage River near Elmore OH (USGS 04195820). This variable was log_10_ transformed.

Cos_DOY is the cosine of the day of the year (D), calculated as 2πD/365. This variable was squared.

## Comprehensive model with continuous monitor data and lagged comprehensive variables (< 2 weeks)

Microcystin_ugL = -23.86 + 0.01502*(LAG_NtoP_ratio) + 2.183*(Leorgn_PH_Ave14d) + 0.1901*(Leorgn_BGA_RFU_Ave24hr) + 0.6934*(Leoc_pH_Ave24hr)

WHERE

Microcystin_ugL is the concentration of microcystin in micrograms per liter. A threshold of 1 μg/L was used for this model.

LAG_NtoP_ratio is the total nitrogen to total phosphorus ratio, determined by a laboratory in a discrete sample collected <2 weeks before the microcystin sample was collected.

Leorgn_PH_Ave14d is the pH measured by the Oregon WTP continuous monitor, averaged over the previous 14 days up to and including 10 a.m. on the day microcystin sample was collected.

Leorgn_BGA_RFU_Ave24hr is the phycocyanin (also called blue-green algae) in relative fluorescence units, measured by the Oregon WTP continuous monitor, averaged over the previous 24 hours up to and including 10 a.m. on the day microcystin sample was collected.

Leoc_pH_Ave24hr is the pH measured by the Ottawa WTP continuous monitor, averaged over the previous 24 hours up to and including 10 a.m. on the day microcystin sample was collected.

## Comprehensive model with continuous monitor data and same day comprehensive variables

Microcystin_ugL = -16.59 + 2.14*(Leorgn_PH_Ave5d) - 44.53*(OrthoP_mgL) + 1.021*(LkLevelChg14day)

WHERE

Microcystin_ugL is the concentration of microcystin in micrograms per liter. A threshold of 1 μg/L was used for this model.

Leorgn_PH_Ave5d is the pH measured by the Oregon WTP continuous monitor, averaged over the previous 3 days up to and including 10 a.m. on the day microcystin sample was collected.

OrthoP_mgL is the concentration of orthophosphate, in milligrams per liter, determined by a laboratory in a discrete sample collected at the same time the microcystin sample was collected.

LkLevelChg14day is the change in lake level at 10:00 a.m. (a typical sampling time) on the day the microcystin sample was collected minus the lake level at 10:00 a.m. 14 days prior, NOAA Marblehead 9063079

# Put-in-Bay recreational site

## Real-time model with continuous monitor data

Microcystin_ugL = -5.169 + 0.6374*(GibIs_pH_Ave14d) + 0.2201*(GibIs_WindSp_ms_Ave14d) + 0.4326*(GibIs_Chlorophyll_RFU_Ave24hr) - 0.004732*(GibIs_SpCond_uScm_Ave7d)

WHERE

Microcystin_ugL is the concentration of microcystin in micrograms per liter. A threshold of 1 μg/L was used for this model.

GibIs_pH_Ave14d is the pH, measured by the Gibraltar Island continuous monitor, averaged over the previous 14 days up to and including 10 a.m. on the day the microcystin sample was collected.

GibIs_WindSp_ms_Ave14d is the wind speed, measured by the Gibraltar island weather station, averaged over the previous 14 days up to and including 10 a.m. on the day the microcystin sample was collected.

GibIs_Chlorophyll_RFU_Ave24hr is the chlorophyll in relative fluorescence units, measured by the Gibraltar island continuous monitor, average of 24 hours up to and including 10 a.m., the time the microcystin sample was collected.

GibIs_SpCond_uScm_Ave7d is the specific conductance, in microsiemens per centimeter, measured by the Gibraltar Island continuous monitor, averaged over the previous 7 days up to and including 10 a.m. on the day microcystin sample was collected

## Comprehensive model with continuous monitor data and lagged comprehensive variables (< 2 weeks)

Microcystin_ugL = 0.4151 - 0.002975*(GibIs_SpCond_uScm_Ave7d) - 0.06414*(TotalFluoro_ugL) + 0.9725*(GibIs_Chlorophyll_RFU_Ave24hr) + 3.092*(Gibs_BGA_logSpC_RATIO_14d)

WHERE

Microcystin_ugL is the concentration of microcystin in micrograms per liter. A threshold of 1 μg/L was used for this model.

GibIs_SpCond_uScm_Ave7d is the specific conductance, in microsiemens per centimeter, measured by the Gibraltar Island continuous monitor, averaged over the previous 7 days up to 10 a.m. on the day microcystin sample was collected.

TotalFluoro_ugL is the Total Fluorescence, in micrograms per liter, measured using the FluoroProbe benchtop reader (bbe-Moldaenke, Kiel, Germany) in a sample collected by use of an integrated tube sampler at 0‒2 m depths.

GibIs_Chlorophyll_RFU_Ave24hr is the chlorophyll, in relative fluorescence units, measured by the Gibraltar Island continuous monitor, averaged over the previous 24 hours up to 10 a.m. on the day microcystin sample was collected.

Gibs_BGA_logSpC_RATIO_14d is the ratio of (1) the phycocyanin (also called blue-green algae) in relative fluorescence units, measured by the Gibraltar Island continuous monitor, averaged over the previous 14 days up to and including 10 a.m., the time the microcystin sample was collected and (2) the specific conductance, in log_10_ microsiemens per centimeter, measured by the Gibraltar Island continuous monitor, averaged over the previous 14 days up to 10 a.m. on the day microcystin sample was collected.
